# Cox-sMBPLS: An Algorithm for Disease Survival Prediction and Multi-Omics Module Discovery Incorporating *Cis*-Regulatory Quantitative Effects

**DOI:** 10.3389/fgene.2021.701405

**Published:** 2021-08-02

**Authors:** Nasim Vahabi, Caitrin W. McDonough, Ankit A. Desai, Larisa H. Cavallari, Julio D. Duarte, George Michailidis

**Affiliations:** ^1^Informatics Institute, University of Florida, Gainesville, FL, United States; ^2^Department of Pharmacotherapy and Translational Research, Center for Pharmacogenomics and Precision Medicine, University of Florida, Gainesville, FL, United States; ^3^Department of Medicine, Indiana University, Indianapolis, IN, United States

**Keywords:** multi-omics, supervised Integration, *cis*-regulatory quantitative, multi-block PLS, survival analysis

## Abstract

**Background:**

The development of high-throughput techniques has enabled profiling a large number of biomolecules across a number of molecular compartments. The challenge then becomes to integrate such multimodal Omics data to gain insights into biological processes and disease onset and progression mechanisms. Further, given the high dimensionality of such data, incorporating prior biological information on interactions between molecular compartments when developing statistical models for data integration is beneficial, especially in settings involving a small number of samples.

**Results:**

We develop a supervised model for time to event data (e.g., death, biochemical recurrence) that simultaneously accounts for redundant information within Omics profiles and leverages prior biological associations between them through a multi-block PLS framework. The interactions between data from different molecular compartments (e.g., epigenome, transcriptome, methylome, etc.) were captured by using *cis*-regulatory quantitative effects in the proposed model. The model, coined Cox-sMBPLS, exhibits superior prediction performance and improved feature selection based on both simulation studies and analysis of data from heart failure patients.

**Conclusion:**

The proposed supervised Cox-sMBPLS model can effectively incorporate prior biological information in the survival prediction system, leading to improved prediction performance and feature selection. It also enables the identification of multi-Omics modules of biomolecules that impact the patients’ survival probability and also provides insights into potential relevant risk factors that merit further investigation.

## Introduction

A key aim in integrating multi-Omics data is to identify combinations of molecular biomarkers that are either predictive of disease onset and outcomes or lead to insights into biological processes and disease mechanisms. To achieve this data integration, it is important to leverage information on interactions/mediations of the different molecular compartments profiled and measured by the various Omics technologies. For example, DNA methylation is known to influence the phenotypic outcome of genetic variation and offers highly complementary information on transcriptional silencing and gene imprinting (Kass et al., 1997). To characterize associations between epigenomics, genomics, and transcriptomics, identification of *cis*-regulatory quantitative effects of SNPs on DNA methylation (meQTL) and mRNA expression (eQTL), and the effect of DNA methylation on mRNA expression (eQTM) has proved particularly informative (Jones, 1999).

In the past decade, a large body of literature was developed to introduce methods relating Omics profiles and disease outcomes, such as recurrence in cancer patients, death, etc. (Park et al., 2002; Tan et al., 2006; Zhang and Zhang, 2020). The most widely used method to model the time to such events is the Cox proportional hazard (Cox-PH) model (Cox, 1972), for which a number of adaptations have been proposed in the literature to make it suitable for use in high-dimensional settings induced by Omics data. Some adaptations leverage various variable selection methods –stepwise approaches, or regularization methods (see Bühlmann et al., 2013 and references therein)-, while others focus on reducing the dimensionality of the predictors by using principal components analysis (PCA) or partial least squares (PLS) (Wold et al., 1983). Partial least squares regression for non-numerical outcome variables was introduced in Garthwaite (1994) and Bastien and Tenenhaus (2001) (PLS-Generalized Linear Regression), and for survival data in (Bastien et al., 2015).

Ridge regression (Hoerl et al., 1975), as the first generation of *L_P_*-regularization methods, utilizes the *L*_2_ – *norm* of the regression coefficients to improve prediction performance. However, ridge regression only shrinks the coefficients toward zero. Instead the Lasso method (Tibshirani, 1996) aims to simultaneously shrink and select a subset of variables through an *L*_1_ – *norm* constraint on the regression coefficients. An important limitation of the lasso method, especially in the case of Omics data, is that lasso tends to select only one variable among a group of correlated variables. For instance, in the multi-Omics framework, there are many features which are interacting as a network (or module) and sharing the same biological pathway. Therefore, the lasso method can poorly indicate this grouping information in the multi-Omics setting. Theoretical and practical explanations of this limitation are given in Efron et al. (2004), and Zou and Hastie (2005). To address these limitations, the elastic-net (Zou and Hastie, 2005) was introduced by imposing a convex combination of the lasso and ridge (*L*_1_, *L*_2_) penalties on the regression coefficients including Cox model. A recent benchmark analysis (Jardillier et al., 2020) of lasso-like penalties (including ridge, lasso, adaptive lasso, and elastic-net) of the Cox model showed a better prediction performance of elastic-net Cox compare to lasso-Cox and adaptive lasso-Cox models.

PLS regression (Wold et al., 1983) has also been used as a dimension reduction method in high-dimensional settings. This method is extended by Garthwaite (1994), and Bastien and Tenenhaus (2001), for generalized regression models (PLS-GLR) and the Cox-PH model as a special case (without considering censoring information). Further developments of PLS-GLR are introduced by Bastien et al. (2005). Chun and Keles̨ (2010), showed that a large number of features in the high-dimensional framework could greatly affect the prediction performance in PLS regressions. They proposed the sparse PLS (sPLS) by incorporating a variable selection constraint directly on the PLS direction vectors (weights). Lee et al. (2013), proposed a new formulation of the sPLS algorithm for survival data. Thereafter, Bastien et al. (2015), proposed a new algorithm called sparse PLS deviance residual (sPLSDR) by use of the normalized martingale residuals as the response variable in the sPLS algorithm.

Random forest, RF (Breiman, 2001), is another powerful prediction system that can consider more complex dependencies between the features. Random survival forest, RSF (Ishwaran et al., 2008), is an extension of random forest RF to analyze survival data (in the presence of right censoring) by introducing a new splitting rule and missing data imputation algorithm. RSF has shown reliable predictions in single-Omics settings (refer to Yosefian et al., 2015 and the references therein). Block Forest (Hornung and Wright, 2019) is another recent extension of RF which considers multiple tuning parameters, where each tuning parameter is associated with one of the data blocks. However, these approaches do not distinguish between variables obtained from different molecular compartments and also ignore any biological constraints –e.g., many variables belong to the same functional pathway, or act as regulators of other variables.

A similar issue arises in complex chemical systems, where variables can be naturally grouped into blocks. Multi-Block PLS, MBPLS (Wangen and Kowalski, 1989) was developed to study association between a numerical outcome variable and blocks of *a priori* defined predictors. The algorithm estimates the model parameters for each block and combines them using the relative importance of each block in predicting the outcome variable. MBPLS has been mostly employed in chemistry, however, multi-Omics data provide a novel opportunity to further extend and apply this algorithm in bioinformatics and genomics (Li et al., 2012).

In this paper, we propose a new integrative survival prediction model named supervised Cox sparse Multi-Block Partial Least Squares (Cox-sMBPLS) by simultaneously controlling the redundancy between Omics profiles from different molecular compartments, focusing on epigenomics, genomics and transcriptomics and incorporating *cis*-regulatory quantitative effects (eQTL, eQTM, meQTL) to integrate additional biological information to the training of the model. Note that the model and integrative strategy are general and can be easily adapted to other molecular compartments with appropriate modifications.

To handle censoring in the survival outcome data, we employ a reweighting technique described in the section “Materials and Methods.” The high dimensionality of the Omics data under consideration is dealt with the use of regularization. The key objective of Cox-sMBPLS is to determine multi-Omics modules [i.e., genes, single-nucleotide polymorphisms (SNPs), and cytosine-phosphate-guanine (CpGs) sites], that are most associated with disease progression and patient’s survival.

## Materials and Methods

**FIGURE 1:**
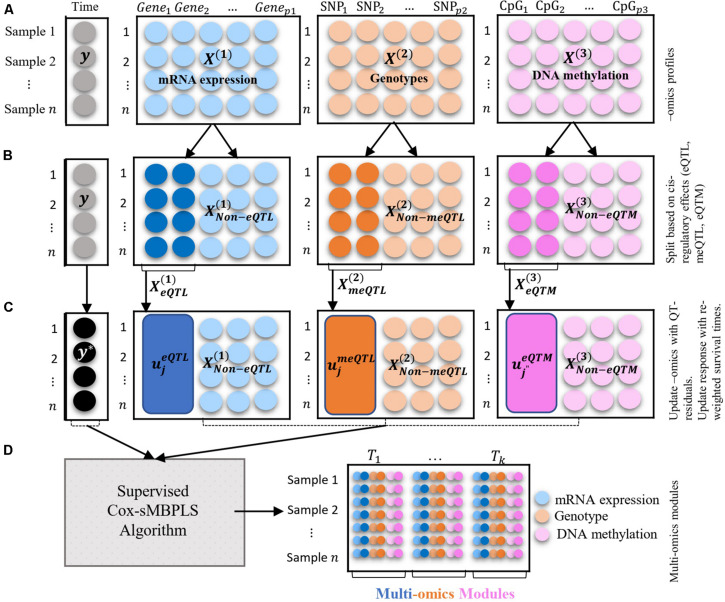
Illustration of the multi-blocks data structure and the supervised Cox-sMBPLS algorithm. **(A)** three blocks of multi-Omics profiles: mRNA expression, genotypes, and DNA methylation. The response variable (**y**) is a vector of survival times of size *n* = 1. **(B)** splitting the Omics blocks based on the *cis*-regulatory quantitative effects (eQTL, eQTM, meQTL). **(C)** updating Omics blocks with QT-residuals, and **y** with reweighted times. **(D)** Multi-Omics modules which are combinations of all three Omics profiles.

An overview of the multi-block data structure used as input, together with how associations between molecular compartments are captured through *cis*-regulatory quantitative effects (eQTL, eQTM, meQTL) to extract low dimensional Omics modules that are predictive of survival times is depicted in Figure 1. As previously mentioned, to handle censoring information on the survival times, we employ an inverse censoring probability weighting scheme to adjust the response variable (observed survival time). The prediction and feature-selection performance of the model is evaluated using various metrics (see parameter tuning and model performance evaluation sub-section).

The rest of the “Materials and Methods” section is as follows: in section “Reweighted Survival Time” we introduce the reweighting of survival time (response variable) using an inverse censoring probability weighting scheme. Doing so we are considering the censoring information in the response variable as well. The reweighted survival time will be used as the response variable in the proposed Cox-sMBPLS algorithm. In section “Partial Least Squares Regression” we briefly introduce the partial least squares regression. In section “Integrating Cis-regulatory Quantitative Effects” we introduce the full process of integrating *cis*-regulatory quantitative effects (eQTL, eQTM, meQTL) and updating Omics-blocks [*X*^(*b*)^, *b* = 1, 2, 3] by regressing out the shared information between QT-pairs (such as, gene-SNP in eQTL) and replacing that with the QT-residuals. In section “Supervised Cox-sMBPLS Algorithm” we provide our proposed supervised Cox-sMBPLS (supervised Cox sparse Multi-Block Partial Least Squares) which uses the reweighted survival time (section “Reweighted Survival Time”) and updated Omics-blocks (section “Integrating Cis-regulatory Quantitative Effects”) as outcome and covariates, respectively. In this section, we first introduce the objective function of supervised Cox-sMBPLS and its solution in the case of univariate response (such as survival time) followed by the detailed algorithm implementation (Algorithm 1). In section “Parameter Tuning and Model Performance Evaluation” we explain the parameter tuning procedure and performance measures which are used to evaluate the feature-selection performance and probability of selecting the correct (important) features for the proposed model. Data sources are fully described in section “Data Source.”

**Algorithm 1. Supervised Cox-sMBPLS algorithm.**

1. Calculating reweighted survival time (y*): Calculate the reweighted survival time as $y_{i}^{*}=\frac{y_{i}.\delta_{i}}{{\hat{S}}_{C}}$, where ${\hat{S}}_{C}$ is the estimated survival function for censoring variable (*C*) and *y_i_* is time to a specific event for the *i*^*th*^ sample, as explained in section “Reweighted Survival Time”.

**2. Incorporating the *cis*-regulatory quantitative effects (eQTL, eQTM, meQTL) information:** Split and update each block employing the corresponding *cis*-regulatory quantitative effects. This process is fully explained in section “Integrating Cis-regulatory Quantitative Effects.”

**3. Computing the latent components and block-weights:** by applying the sMBPLS algorithm on *X*^(*b*)^ (as independent variables) and *y*^∗^ (as dependent variable) as follows:

do for *k* = 1, …, *K* where *k* (a tuning parameter) is the number of the latent components,

3.1 Set ${\hat{\beta }}_{PLS}^{\left(b\right)}=0$, Ω^(*b*)^ = {}, **y** = y* and *k* = 1, where *b* = 1, 2, 3.
3.2 ${\text{w}}^{\left(b\right)}=soft-threshold\left(\frac{{\overset{´}{X}}^{\left(b\right)*}y^{*}}{{||{\overset{´}{X}}^{\left(b\right)*}y^{*}||}_{2}},\lambda \right)$, where λ is a non-negative sparsity parameter.
3.3 τ^(*b*)^ = *X*^(*b*)^**w**^(*b*)^, *b* = 1, 2, 3
3.4 τ = [τ^(1)^, τ^(2)^, τ^(3)^]
3.5 $\omega ={\overset{´}{\text{y}}}^{*}\tau$
3.6 $\text{T}=\tau \overset{´}{\omega }$
3.7 Update Ω^(*b*)^ as $\left\{j^{\left(b\right)}:{\hat{w}}_{j^{\left(b\right)}}^{\left(b\right)}\neq 0\right\}\cup \left\{j^{\left(b\right)}:{\hat{\beta }}_{PLS_{j^{\left(b\right)}}}^{\left(b\right)}\neq 0\right\}$, where *j*^(*b*)^ ∈ {1, …, *p*_*b*_}
3.8 Fit PLS regression to *X*^(*b*)*^ (updated based on Ω^(*b*)^) and y* in each block (*b* = 1, 2, 3) using *k* number of latent components.
3.9 Update ${\hat{\beta }}_{PLS}^{\left(b\right)}$ with new estimated coefficients resulted from the PLS regression in step 3.8. Update y* as ${\text{y}}^{*}\to {\text{y}}^{*}-X^{\left(b\right)*}{\hat{\beta }}_{PLS}^{\left(b\right)}$

**4. Final Cox-sMBPLS model:** Fit a Cox-PH model with (*y*_*i*_, δ_*i*_) and remaining latent components from the sMBPLS algorithm.

### Reweighted Survival Time

Let ${\tilde{y}}_{i}$ and *C_i_* indicate the independent true survival time and censoring time for the *i*^*th*^ subject (*i* = 1, …, *n*), respectively. The observed data consist of pairs {(*y*_*i*_, δ_*i*_)|*i* = 1, …, *n*} where $y_{i}=min\left({\tilde{y}}_{i},C_{i}\right)$ is the observed survival time and $\delta_{i}=I\left({\tilde{y}}_{i}\leq C_{i}\right)$ with I(.) denoting the indicator function. Note that ${\tilde{y}}_{i}=y_{i}$ if and only if δ_*i*_ = 1 (i.e., the subject is uncensored). To deal with the right censoring in the MBPLS algorithm, we use the reweighting method (Datta, 2005) to construct the so-called *adjusted observed survival time* using inverse censoring probability weighting. To briefly summarize the reweighting procedure, let *S*_*C*_ be the survival function of the censoring variable *C*, i.e., *S*_*C*_(*t*) = *P*(*C* > *t*), *t* ≥ 0. Then,

$$\begin{array}{c} E\left(\frac{\delta_{i}y_{i}}{S_{C}\left(y_{i}-\right)}\right)=E\left(E\left(\frac{\delta_{i}{\tilde{y}}_{i}}{S_{C}\left({\tilde{y}}_{i}-\right)}|{\tilde{y}}_{i}\right)\right)\\ \;=E\left(\frac{{\tilde{y}}_{i}}{S_{C}\left({\tilde{y}}_{i}-\right)}E\left(I\left(C_{i}\geq {\tilde{y}}_{i}\right)|{\tilde{y}}_{i}\right)\right)=E\left({\tilde{y}}_{i}\right) \end{array}$$

This identity can be taken as the theoretical basis for estimating the mean observed survival time by the (weighted) sample average $\mu =\left(\sum_{i=1}^{n}{\tilde{w}}_{i}y_{i}\right)/n$, where the weights are ${\tilde{w}}_{i}=0$ for a censored survival time and ${\tilde{w}}_{i}={\left({\hat{S}}_{C}\left(y_{i}-\right)\right)}^{-1}$ for an observed survival time. Here, *S_C_* will be calculated using the intercept-only Cox-PH model (Kaplan-Meier) with the status indicator 1−δ_*i*_ (using *survfit* R function)^1^.

### Partial Least Squares Regression

Partial least squares (PLS) regression, originally introduced by Wold et al. (1983), has been applied in various ill-conditioned linear regression models as both dimension reduction and inference tool. PLS regression works under the assumption of basic latent-decomposition of both *X* (a *n* × *p* matrix of covariates) and *y* (a *n* × 1 matrix of response variable in a univariate case). In contrast to PCA, PLS uses both *X* and *y* to construct the latent components (*T = XW*) by maximizing a successive maximization problem. The objective function to find the weight vectors (*W* = (**w**_1_, …, **w**_*k*_) is as follows:

$$\begin{array}{c} argmax_{w}\overset{´}{\text{w}}\overset{´}{X}y\overset{´}{y}X\text{w},fork=1,\ldots ,K\\ s.t.{||w||}_{2}=1, \end{array}$$

where *k* is the number of the latent components (tuning parameter or fixed by user), and *W* is a *p* × *k* matrix of weights (also called direction vector). The latent component *T* (a *n* × *k* matrix) is then calculated as *T* = *XW*.

### Integrating Cis-Regulatory Quantitative Effects

Genome-wide quantitative trait loci (QTL) mapping enables the determination of genetic loci affecting other Omics, i.e., transcriptome, proteome, and metabolome. Some Omics markers do not directly contribute to the phenotype and affect the disease through other intermediates-Omics. Therefore, considering these QTL associations in each Omics layer can provide more functional information about disease-associated markers. In fact, a QTL-pair is a pair of different Omics (for instance, SNP-CpG in eQTL) that are (highly) associated regarding their effects on the underlying disease (outcome). Hence involving both elements of a QTL-pair (i.e., SNP and gene in an eQTL-pair) in a regression model will not add much more information than involving only one of these elements (at the cost of degrees of freedom and multi-collinearity). One way around this is to consider one element of a QTL-pair in the regression model (such as SNP in an eQTL-pair), then regress out the effect of this element and replace the second element with the residuals. This way, we are considering unwanted/uncorrelated information besides the QTL information in the regression model, avoiding multicollinearity.

As shown in Figure 3, for the eQTL-pairs (SNP-gene pairs), we keep the SNPs and replace the genes with residuals by regression out the effect of the SNPs. For the meQTL-pairs (SNP-CpG pairs), we keep the actual DNA methylations (CpGs) and update the genotypes (SNPs) with residuals by regression out the effect of the CpGs. For the eQTM-pairs (gene-CpG pairs) we keep the actual gene expression and update the methylations (CpGs) with residuals by regression out the effect of the genes. The residuals are obtained using the univariate linear regression models. The details of updating each Omics block using QTL-residual are as follows.

Let $X^{\left(b\right)}=X_{n\times p_{b}}^{\left(b\right)}$ denotes the observed data in block *b* (*b* = 1, …, *B*), a matrix of *p_b_* features/covariates measured for *n* samples. For ease of presentation, we consider the following *B* = 3 blocks (mRNA expression, genotypes, DNA methylation). However, the proposed modeling framework can accommodate a larger number of blocks and thus other Omics types (such as proteomics and metabolomics) with minor modifications. Thus, let $X_{n\times p_{1}}^{\left(1\right)},X_{n\times p_{2}}^{\left(2\right)},X_{n\times p_{3}}^{\left(3\right)}$ denote blocks of mRNA expression of *p*_1_ genes, genotypes of *p*_2_ SNPs, and DNA methylation of *p*_3_ CpGs for *n* samples, respectively. Next, we split each *X*^(*b*)^ based on prior biological knowledge gleaned from eQTL, meQTL, eQTM information. **The first block** (mRNA expression) is split by utilizing the eQTL information as follows:

$$X_{n\times p_{1}}^{\left(1\right)}=\left[X_{n\times q_{1}}^{\left(1\right)}X_{n\times \left(p_{1}-q_{1}\right)}^{\left(1\right)}\right]=\left[X_{eQTL}^{\left(1\right)}X_{Non-eQTL}^{\left(1\right)}\right],$$

where *q*_1_ is the number of the eQTL-genes (e.g., with adjusted *P*-value < 0.05). Whole blood eQTL data are extracted from the Genotype-Tissue Expression Project (GTEx) (Lonsdale et al., 2013) that are used to extract eQTL-genes and their eQTL-SNP pairs. Specifically, suppose that $SNP_{j}^{eQTL}$ is a vector of SNPs which are in eQTL with (single) *gene_j_* (*j* = 1, …, *q*_1_) and $SNP_{j}^{eQTL}\in X^{\left(2\right)}$. By regressing out the effect of these SNPs, the so-called eQTL residuals ($u_{j}^{eQTL}$) are defined as the residuals of the regression of the *j*^*th*^ eQTL-gene ($\in X_{eQTL}^{\left(1\right)}$) on $SNP_{j}^{eQTL}$, as follows:

$$gene_{j}\propto \sum_{j=1}^{q_{1}}\alpha SNP_{j}^{eQTL}+u_{j}^{eQTL},wheregene_{j}\in X_{eQTL}^{\left(1\right)}$$

We then update $X_{n\times p_{1}}^{\left(1\right)}$ by replacing $X_{eQTL}^{\left(1\right)}$ with $u_{j}^{eQTL}$. Therefore, the updated $X_{n\times p_{1}}^{\left(1\right)}$becomes:

$$X_{n\times p_{1}}^{\left(1\right)*}=\left[u_{j}^{eQTL}X_{Non-eQTL}^{\left(1\right)}\right].$$

**The second block** (genotypes) is split by utilizing the meQTL information as follows:

$$X_{n\times p_{2}}^{\left(2\right)}=\left[X_{n\times q_{2}}^{\left(2\right)}X_{n\times \left(p_{2}-q_{2}\right)}^{\left(2\right)}\right]=\left[X_{meQTL}^{\left(2\right)}X_{Non-meQTL}^{\left(2\right)}\right],$$

where *q*_2_ is the number of the meQTL-SNPs (with adjusted *P*-value < 0.05). Whole blood *cis*-meQTL data were extracted from BIOS QTL (Zhernakova et al., 2017) that are used to extract the meQTL-SNPs and their meQTL-CpG pairs. Specifically, suppose that $CpG_{\overset{´}{ȷ}}^{meQTL}$ is a vector of CpGs which are in meQTL with (single) ${\mathrm{SNP}}_{\overset{´}{ȷ}}\;(\overset{´}{ȷ}=1,\ldots ,q_{2})$ and $CpG_{\overset{´}{ȷ}}^{meQTL}\in X^{\left(3\right)}$. By regressing out the effect of these CpGs, the so-called “meQTL residuals” ($u_{\overset{´}{ȷ}}^{meQTL}$) are defined as the residuals of the regression of ${\overset{´}{ȷ}}^{th}$ meQTL-SNP ($\in X_{meQTL}^{\left(2\right)}$) on $CpG_{\overset{´}{ȷ}}^{meQTL}$, as follows:

$$snp_{\overset{´}{ȷ}}\propto \sum_{\overset{´}{ȷ}=1}^{q_{2}}\overset{´}{\alpha }cpg_{\overset{´}{ȷ}}^{meQTL}+u_{\overset{´}{ȷ}}^{meQTL},wheresnp_{\overset{´}{ȷ}}\in X_{meQTL}^{\left(2\right)}.$$

We then update $X_{n\times p_{2}}^{\left(2\right)}$ by replacing $X_{meQTL}^{\left(2\right)}$with $u_{\overset{´}{ȷ}}^{meQTL}$. Therefore, updated $X_{n\times p_{2}}^{\left(2\right)}$becomes:

$$X_{n\times p_{2}}^{\left(2\right)*}=\left[u_{\overset{´}{ȷ}}^{meQTL}X_{Non-meQTL}^{\left(2\right)}\right].$$

**The third block** (DNA methylations) is split by utilizing the eQTM information as follows:

$$X_{n\times p_{3}}^{\left(3\right)}=\left[X_{n\times q_{3}}^{\left(3\right)}X_{n\times \left(p_{3}-q_{3}\right)}^{\left(3\right)}\right]=\left[X_{eQTM}^{\left(3\right)}X_{Non-eQTM}^{\left(3\right)}\right],$$

where *q*_3_ is the number of the eQTM-CpGs (with adjusted *P*-value < 0.05). Whole blood *cis*-eQTM data were extracted from BIOS QTL (Zhernakova et al., 2017) that are used to extract the eQTM-CpGs and their eQTm-gene pairs. Specifically, suppose that $gene_{j^{"}}^{eQTM}$ is a vector of genes which are in eQTM with (single) CpG *j*^"^ (*j*^"^ = 1, …, *q*_3_). By regressing out the effect of these genes, the so-called “eQTM residuals” ($u_{j^{"}}^{eQTM}$) are defined as the residuals of the regression of the *j*^"*th*^ eQTM-CpG ($\in X_{eQTM}^{\left(3\right)}$) on $gene_{j^{"}}^{eQTM}$, as follows:

$$cpg_{j^{"}}\propto \sum_{j^{"}=1}^{q_{3}}\alpha^{"}gene_{j^{"}}^{eQTM}u_{j^{"}}^{eQTM},wherecpg_{j^{"}}\in X_{eQTM}^{\left(3\right)}.$$

We then update $X_{n\times p_{3}}^{\left(3\right)}$ by replacing $X_{eQTM}^{\left(3\right)}$ with $u_{j^{"}}^{eQTM}$. Therefore, updated $X_{n\times p_{3}}^{\left(3\right)}$becomes:

$$X_{n\times p_{3}}^{\left(3\right)*}=\left[u_{j^{"}}^{eQTM}X_{Non-eQTM}^{\left(3\right)}\right].$$

The illustration of the multi-Omics data structure and Omics-block updating procedure is presented in Figure 1.

### Supervised Cox-sMBPLS Algorithm

Let *X*^(*b*)^, *b* = 1, 2, 3 (*X*^(*b*)^ = *X*^(*b*)*^ as explained in section “Integrating Cis-regulatory Quantitative Effects”) and **y** (**y** = y* as explained in section “Reweighted Survival Time”) be the covariate matrices (blocks) and response vector on the same *n* samples, respectively. In each block, the dimensionality of the block can be reduced by taking a linear combination of the covariates τ^(*b*)^ = *X*^(*b*)^**w**^(*b*)^, where **w**^(*b*)^ is direction vector (also called weight vector) that express the importance of each covariate on the latent component τ^(*b*)^ (a *n*×1 matrix). Li et al. (2012), suggested using a weighted sum of the latent components over the blocks as the combined latent component. Therefore, we define $\tau =\sum_{b=1}^{3}\tau^{\left(b\right)}\omega^{\left(b\right)}$ as the combined latent component, where ω^(*b*)^ (ω^(*b*)^ > 0) is the weight for block *b*, which indicates the contribution of this block to the covariance structure of the input and response (**y**) data. We can then posit the following optimization problem for calculating the latent components across all blocks:

(1) $$\begin{array}{c} \max_{w^{\left(b\right)}}cov\left(\tau ,y\right),with\tau =\sum_{b=1}^{3}\tau^{\left(b\right)}\omega^{\left(b\right)},and\tau^{\left(b\right)}=X^{\left(b\right)}{\text{w}}^{\left(b\right)},\\ \;\omega^{\left(b\right)}\in {\mathbb{R}}^{+},X^{\left(b\right)}\in {\mathbb{R}}^{n\times p_{b}},{\text{w}}^{\left(b\right)}\in {\mathbb{R}}^{p_{b}},\text{y}\in {\mathbb{R}}^{n},\\ \;subjectto{||{\text{w}}^{\left(b\right)}||}_{2}={||\omega ||}_{2}=1. \end{array}$$

A straightforward extension of problem (1) to the sparse version could be obtained by adding an *L*_1_ penalty on direction vector **w**^(*b*)^; i.e., ||**w**^(*b*)^||_1_ ≥ λ, for some positive tuning parameter λ. However, Jolliffe et al. (2003), showed, via an example, that this formulation may not lead to a sufficiently sparse solution. The sparsity issue for PCA was first considered by Zou et al. (2006) by imposing both *L*_1_&*L*_2_ constraints on the weight coefficients.

In the case of PLS, Chun and Keles̨ (2010) used the same approach by imposing an *L*_1_ constraint onto a surrogate of the direction vector. Therefore, a generalized version of the optimization problem using a combined *L*_1_&*L*_2_ regularization component becomes:

(2) $$\begin{array}{c} \min_{w^{\left(b\right)},c^{\left(b\right)}}\{-\kappa \omega^{{\left(b\right)}^{2}}{\overset{´}{\text{w}}}^{\left(b\right)}Z^{\left(b\right)}{\text{w}}^{\left(b\right)}+\omega^{{\left(b\right)}^{2}}\left(1-\kappa \right){\left(c^{\left(b\right)}-{\text{w}}^{\left(b\right)}\right)}^{\prime }Z^{\left(b\right)}\\ \;\left(c^{\left(b\right)}-{\text{w}}^{\left(b\right)}\right)+\lambda_{1}||c^{\left(b\right)}|{|}_{1}+\lambda_{2}||c^{\left(b\right)}|{|}_{2}\},\\ \;subjectto{||{\text{w}}^{\left(b\right)}||}_{2}={||\omega ||}_{2}=1. \end{array}$$

For any non-negative λ_1_ and λ_2_. $Z^{\left(b\right)}={\overset{´}{X}}^{\left(b\right)}\text{y}\overset{´}{\text{y}}X^{\left(b\right)}$, *c*^(*b*)^ is the surrogate of the direction vector in block *b*, which is kept close to the original direction vector **w**; and κ is a tuning parameter. κ is the concave-penalty parameter to control the amount of the weight is given to the concave part of the objective function $[{\overset{´}{\text{w}}}^{\left(b\right)}Z^{\left(b\right)}{\text{w}}^{\left(b\right)}$], and therefore, to control the local-solution issue. For more details and history of recasting from equation (1) to equation (2), see Supplementary Section 1.1.

Bastien et al. (2015) also employed this method to propose a sparse PLS for censored data. Chun and Keles̨ (2010) solved problem (2) by alternatively iterating between solving for **w** after fixing *c* and solving for *c* for fixed **w**. However, they showed that in the case of a univariate response, problem (2) does not depend on and often needs a large λ_2_ value to be solved. Therefore, we use λ_2_ → ∞ which yields the special case of the elastic-net regularization, called univariate soft-thresholding (Zou and Hastie, 2005). Hence, the solution to problem (2) has the following closed form:

(3) $${\hat{c}}^{\left(b\right)}={\left(\frac{\overset{´}{X^{\left(b\right)}}\text{y}}{||\overset{´}{X^{\left(b\right)}}\text{y}||}-\frac{\lambda }{2}\right)}_{+}sign\left(\frac{\overset{´}{X^{\left(b\right)}}\text{y}}{||\overset{´}{X^{\left(b\right)}}\text{y}||}\right),\lambda \geq 0$$

where $\frac{\overset{´}{X^{\left(b\right)}}y}{||\overset{´}{X^{\left(b\right)}}y||}$ is the first direction vector, and $soft-threshold\left(\Phi ,\lambda \right)=\left(\Phi -\frac{\lambda }{2}\right)sign\left(\Phi \right)$ is the soft-thresholding operator with a fixed non-negative parameter λ (λ = λ_1_). The algorithm is then followed by a PLS regression on the selected variables, and iterated with updating the response variable, **y**. The proof for the solution in equation (3) is provided in Supplementary Section 1.1. The full algorithm is described below.

The conjugacy of direction vectors (similar to orthogonality issue in PCA-kind problems) is addressed by keeping the Krylov subsequence structure of the direction vectors in a restricted *X*-space of selected variables (*X*^(*b*)^ ∈ Ω^(*b*)^) (Chun and Keles̨, 2010). Specifically, at each step of the Algorithm 1, it searches for relevant variables, the so-called active variables (updated in step 3.7), by optimizing equation (2) and updates all direction vectors to form a Krylov subsequence on the subspace of the active variables. This is simply achieved by conducting PLS regression by using the selected variables (see step 3.8, Algorithm 1).

Initial values are set in step 3.1 and sparse weight (direction) vectors are calculated in step 3.2 where λ is a non-negative (sparsity) tuning parameter which is tuned using a k-fold cross-validation (see section “Parameter Tuning and Model Performance Evaluation” for details). In step 3.3 the latent components for each block are calculated ($\tau_{n\times 1}^{\left(1\right)},\tau_{n\times 1}^{\left(2\right)},\tau_{n\times 1}^{\left(3\right)}$) which are then combined using a weighted sum over the blocks to calculate the combined latent component (**T**_*n*×1_) in step 3.6. Blocks’ weight (ω) are calculated in Step 3.5. The so-called active variables set (Ω^(*b*)^) is then updated in step 3.7 followed by a PLS regression using active variables X^*(*b*)^ ∈ Ω^(*b*)^ as covariates and y* as response variable. The PLS regression is fit using *wpls* R function, adapted from *spls* R package^2^. Response variable (y*) is then updated in step 3.9. Note that $\overset{´}{X^{\left(b\right)*}}$ is scaled, including genotype data (categorical variable), as suggested by Tibshirani (1997). To scale the categorical genotypes [$\overset{´}{X^{\left(2\right)*}}$], we considered the fact that encoded genotypes (0, 1, 2) are quantitative measures correspond to the number of minor alleles in the genotype. Latent components are computed using the updated data, *X*^(*b*)*^ and y* (step 3.9). The solution to the optimization function (2) also enables us to identify multi-Omics modules. These modules are linear combinations of multiple Omics profiles with large absolute values of **w**^(*b*)^ if happen together. It is possible to apply different sparsity parameters for each block and or direction vector, which is avoided here due to the computational burden of tuning multiple parameters. The illustration of the data structure and the supervised Cox-sMBPLS algorithm is provided in Figure 1.

### Parameter Tuning and Model Performance Evaluation

Cross-validation (CV) is used to tune the number of components (*k*) and the sparsity (λ) hyper-parameters that lead to the best prediction performance. In principle, we can try different combinations of *k* (number of the latent components) and λ (sparsity). The chosen *k* and λ are the ones giving the highest model performance measures. For the real data analysis, we considered $1=k=min\left\{p,\frac{\nu -1}{\nu }n\right\}$ where *p* is the total number of the covariates, ν is the fold number in the (k-fold) CV, and *n* is the sample size. This upper bound for the number of the latent components is suggested by Chung et al. (2012). In each iteration, the supervised Cox-sMBPLS model is trained using a training-set (in the numerical work, we set it to 80% of data). The test data-set (remaining 20% of data) is then used to evaluate the predictive performance using Harrell’s C-index (Harrell et al., 1982) (∑ijIi(y^i=y^j)I(yi=yj)∑ijIi(yi=yj)) and time-specific area under the ROC-curve, AUC. We used the incident/dynamic (I/D) ROC-curves (Heagerty and Zheng, 2005) and Uno (Uno et al., 2007) and Chambless (Chambless and Diao, 2006) estimators of cumulative/dynamic (C/D) AUC (more information is provided in Supplementary Section 1.2).

### Data Source

The analyzed data set contains information on 91 subjects with heart failure (HF); namely, with preserved ejection fraction (HFpEF) and reduced ejection fraction (HFrEF). Further, 47% of them experienced death or a hospitalization event. The original discovery cohort included 103 HF (HFpEF and HFrEF) patients with complete data of all Omics types (mRNA expression, genotypes, and DNA methylation), 12 of which were removed due to sex mismatch and *n* = 91 patients remained in the analysis. The subjects were recruited from cardiology clinics during a four-year period (2011–2015) at the University of Illinois at Chicago (UIC). All patients provided written, informed consent (Mansour et al., 2016; Duarte et al., 2018).

RNA profiles were obtained by using the Affymetrix Human Gene 2.0 ST array. After quality control procedures, 27 645 genes were kept for subsequent analysis. Genotypes were measured by high-density genome-wide bead array genotyping (Affymetrix Axiom PanAfrican Array). We excluded SNPs with a missing rate ≥10%, monomorphic SNPs with MAF < 0.01% and SNPs on the negative strands. Additional genotypes were imputed based on a two-step approach. First, the samples were phased into a series of estimated haplotypes, and then, imputation was performed on them. After imputation, genotypes with *R*^2^ = 80% were excluded to keep only high-quality imputed profiles. We then performed linkage disequilibrium pruning (LDP). Thereafter, whole blood *cis*-eQTL data from the Genotype-Tissue Expression Project (GTEx) (Lonsdale et al., 2013) were used to remove non-eQTL-SNPs with adjusted eQTL-pvalue > 0.1. We applied this filter as part of the pre-analysis feature selection procedure since it is shown that GWAS eQTL-SNPs tended to be more significant compared to non-eQTL-SNPs (Gorlov et al., 2019). In total, 578 846 SNPs remained in the study. DNA methylation profiles were measured using the Illumina Infinium Human Methylation 450 (450K) BeadChip array. Whole blood *cis*-eQTM data from BIOS QTL (Zhernakova et al., 2017) were then utilized to remove the non-eQTM-CpGs with eQTM-pvalue > 0.1. In total, 12 283 CpGs remained in the study.

## Results

### Results on Simulated Data

We performed a set of simulation studies in order to evaluate the prediction accuracy of the proposed supervised Cox-sMBPLS model. The settings under consideration aim to control the redundancy within the Omics profiles (via a soft-threshold), the association between the Omics profiles (via *cis*-regulatory quantitative effects), and the relevance of each Omics profile or a combination of them to explain the survival probabilities. We compared the proposed model to elastic-net Cox-PH (El-net Cox) (Simon et al., 2011), random survival forest (RSF) (Ishwaran et al., 2008), Block Forest (Hornung and Wright, 2019), and multiple co-inertia analysis (MCIA) (Min and Long, 2020). All models are trained on 80% of the samples and tested on the left-out 20% portion. Further, cross-validation (CV) is used to tune the hyperparameters for all methods considered. To generate more realistic samples of the Omics profiles, we randomly sampled from a real-world multi-Omics data set, which is described in the next section. We simulated 10 800 replicates based on combinations of the following factors for a total of 9 scenarios: the number of the latent components *k*, the censoring rate δ the number of blocks *b*, and the number of features *p_b_* in block *b*. Details on the simulation settings are provided in Table 1.

**TABLE 1:** Simulation settings based on the different number of latent components (*k*), censoring rate (δ) and *p_b_* number of features in block *b*.

Scenario #	Censoring	Dimensionality	Number of Components
1**	δ = 10% (Low)	*p*_*b*_ = 100 = υ^(*b*)^* (Low)	*k* = 2, 5,10
2		*p*_*b*_ = 1000 = υ^(*b*)^ (Moderate)	*k* = 2, 5,10
3		*p*_*b*_ = 10 000 = υ^(*b*)^ (High)	*k* = 2, 5,10
4	δ = 40% (Moderate)	*p*_*b*_ = 100 = υ^(*b*)^* (Low)	*k* = 2, 5,10
5		*p*_*b*_ = 1000 = υ^(*b*)^ (Moderate)	*k* = 2, 5,10
6		*p*_*b*_ = 10 000 = υ^(*b*)^ (High)	*k* = 2, 5,10
7	δ = 60% (High)	*p*_*b*_ = 100 = υ^(*b*)^* (Low)	*k* = 2, 5,10
8		*p*_*b*_ = 1000 = υ^(*b*)^ (Moderate)	*k* = 2, 5,10
9		*p*_*b*_ = 10 000 = υ^(*b*)^ (High)	*k* = 2, 5,10

Three -Omics blocks, b = 3, (mRNA expression, genotypes and DNA methylation) sampled from the same n = 91 samples are considered. * We defineυ^(b)^, as the weight of each block, relative to the total number of genes: $\upsilon^{\left(1\right)}=\frac{27 645}{27 645}=1$, $\upsilon^{\left(2\right)}=\frac{578 846}{27 645}=20.9$, $\upsilon^{\left(3\right)}=\frac{12 283}{27 645}=0.4$. ** 1350 independent replicates for each scenario, with 450 replicates for each k (k = 2, 5, 10) in scenarios with low and moderate levels of dimensionality, and 300 replicates for each k in the scenario with a high level of dimensionality.

**FIGURE 2:**
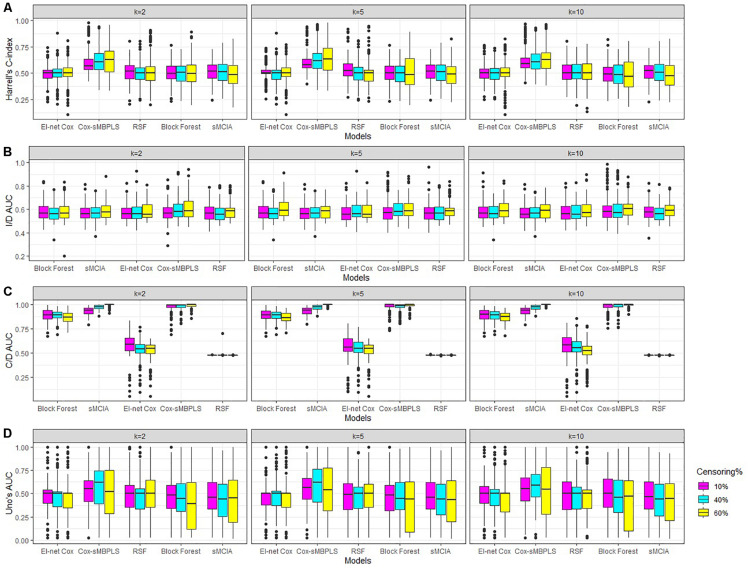
Simulations results for scenarios with a low level of dimensionality. Boxplots for **(A)** Harrell’s C-index, **(B)** I/D AUC, **(C)** C/D AUC, and **(D)** Uno’s AUC values. Results are shown for different censoring rates (δ = 10,40, 60%) and number of components (*k* = 2, 5, 10).

We sample true predictor matrices $X_{T}^{b}$ (*b* = 1, 2, 3) of dimension *n = p_b_*, with fixed sample size *n* = 91. Matrices $X_{T}^{b}$ (*b* = 1, 2, 3) are random samples (without replacement) from the real Omics data presented in the next section. For the construction of true latent components (τ_*T*_), we assume that some of the features in each block have small or no effect on the response variable by specifying sparse (true) direction vector (**w**_*T*_). These weights are sampled from the distribution of the weights collected from a standard sparse PLS on a random sample of the Omics data. Therefore, true latent components are sparse across all simulations. The response variable (*y*_*i*_, δ_*i*_) for sample *i* (*i* = 1, 91) is simulated using a flexible-hazard model (Harden and Kropko, 2019). We simulate the replicates using twenty different seed numbers and the results assess the stability of the models against the seed numbers. More details, including the simulation algorithm are provided in Supplementary Section 2. The results of the simulation studies for the low level of dimensionality are shown in Table 2 and Figure 2 (for *k* = 2, 5). See Supplementary Table 1 for the full results of the low level of dimensionality (including *k* = 10). The results of the moderate and high levels of dimensionality are presented in Supplementary Tables 2, 3) and are very similar to the results of the low level of dimensionality.

**TABLE 2:** Simulation results for the scenarios with a low level of dimensionality (total of 2230 features, and *n* = 91).

Censoring %	Measure	Number of components									
		k = 2					k = 5				
		Cox-sMBPLS	El-net Cox	RSF	Block forest	MCIA	Cox-sMBPLS	El-net Cox	RSF	Block forest	MCIA
10% (Low)	C-index	**0.60** (0.10)	0.49 (0.08)	0.50 (0.10)	0.49 (0.09)	0.51 (0.09)	**0.60** (0.09)	0.51 (0.07)	0.53 (0.10)	0.51 (0.10)	0.51 (0.09)
	C/D AUC*	**0.95** (0.13)	0.38 (0.30)	0.46 (0.06)	0.87 (0.13)	0.91 (0.12)	**0.97** (0.09)	0.36 (0.29)	0.46 (0.09)	0.87 (0.12)	0.91 (0.12)
	I/D AUC**	**0.58** (0.07)	0.57 (0.06)	**0.58** (0.07)	0.57 (0.07)	0.57 (0.07)	**0.58** (0.08)	0.57 (0.07)	0.57 (0.08)	0.57 (0.07)	0.57 (0.07)
	Uno’s AUC***	**0.52** (0.22)	0.46 (0.18)	0.48 (0.22)	0.46 (0.23)	0.49 (0.23)	**0.53** (0.23)	0.44 (0.18)	0.46 (0.23)	0.46 (0.23)	0.45 (0.23)
40% (Moderate)	C-index	**0.62** (0.11)	0.49 (0.09)	0.51 (0.10)	0.49 (0.10)	0.51 (0.11)	**0.63** (0.11)	0.49 (0.09)	0.51 (0.10)	0.49 (0.11)	0.51 (0.11)
	C/D AUC	**0.94** (0.18)	0.35 (0.27)	0.39 (0.19)	0.84 (0.19)	0.90 (0.20)	**0.94** (0.17)	0.36 (0.27)	0.37 (0.19)	0.84 (0.19)	0.90 (0.20)
	I/D AUC	**0.60** (0.08)	0.57 (0.07)	0.57 (0.08)	0.56 (0.07)	0.57 (0.07)	**0.60** (0.07)	0.58 (0.07)	0.58 (0.08)	0.57 (0.07)	0.57 (0.07)
	Uno’s AUC	**0.54** (0.29)	0.44 (0.22)	0.45 (0.23)	0.44 (0.25)	0.42 (0.24)	**0.56** (0.28)	0.45 (0.22)	046 (0.23)	0.45 (0.25)	0.42 (0.24)
60% (High)	C-index	**0.63** (0.13)	0.50 (0.10)	0.51 (0.13)	0.49 (0.14)	0.48 (0.13)	**0.64** (0.12)	0.50 (0.10)	0.50 (0.12)	0.50 (0.16)	0.48 (0.12)
	C/D AUC	**0.93** (0.23)	0.30 (0.27)	0.35 (0.21)	0.81 (0.20)	**0.93** (0.24)	**0.93** (0.23)	0.29 (0.26)	0.34 (0.21)	0.82 (0.20)	**0.93** (0.24)
	I/D AUC	**0.61** (0.09)	0.58 (0.07)	0.59 (0.06)	0.57 (0.09)	0.58 (0.07)	0.60 (0.08)	0.59 (0.07)	0.59 (0.06)	**0.61** (0.09)	0.58 (0.07)
	Uno’s AUC	**0.50** (0.31)	0.45 (0.24)	0.47 (0.27)	0.40 (0.28)	0.41 (0.29)	**0.51** (0.32)	0.45 (0.24)	0.46 (0.27)	0.40 (0.30)	0.41 (0.29)

Performance measures are averaged over S = 450 simulations. SDs are shown in the parentheses. * Chambless estimator of cumulative/dynamic (C/D) AUC. ** Incident/dynamic (I/D) AUC. *** Uno estimator of cumulative/dynamic (C/D) AUC. Bold values show the highest performance for each measurement and k.

The proposed supervised Cox-sMBPLS model exhibited better performance than El-net Cox, RSF, Block forest and MCIA models in both scenarios with low and moderate level of dimensionality. The prediction performance (C-index) and feature-selection performance (AUCs) of the proposed Cox-sMBPLS model remained higher than other models regardless of the different changing parameters. When increasing the censoring rate from 10 to 60%, the feature-selection performance (C/D AUC) of all models decreased (except for MCIA) (Table 2): in Cox-sMBPLS decreased by 2% for *k* = 2, 4% for *k* = 5 and *k* = 10; in El-net Cox decreased by 8%, 7%, and 11% for *k* = 2, 5, 10, respectively; in RSF decreased by 11%, 3%, and 10% for *k* = 2, 5, 10, respectively; in Block Forest decreased by 6%, 5%, and 5% for *k* = 2, 5, 10, respectively; in MCIA decreased by 3% and 0% for *k* = 2, 5, respectively, and increased by 2% for *k* = 10. Amongst these models, the proposed Cox-sMBPLS (2–4% decrease) and Block Forest (5–6% decrease) are more stable than El-net Cox (7-11% decrease), RSF (3–11% decrease), and MCIA (0–3% decrease and/or 2% increase) against the censoring rate. The feature selection results showed less stability against censoring rate using other performance measures (I/D AUC, and Uno’s AUC). I/D AUC, and Uno’s AUC tended to increase while increasing the censoring rate. This may be due to the relatively low number of events and the following low number of patient-pairs used to estimate the measures. Rahman et al. (2017) experienced the same results in an extensive simulation study comparing the different performance measures for survival models. In general, the results were more stable against censoring rate by increasing the number of the latent components, *k* (see Supplementary Tables 1–3). In most of the settings, the variability (SDs) of all measures increased by increasing the censoring rate, as expected. Similar results for other settings are presented in Supplementary Tables 2, 3 and Supplementary Figures 1, 2. When increasing the dimensionality of the predictors, the performance of all models decreased, even though the Cox-sMBPLS model continued to outperform the other ones. Based on the AUC values, Cox-sMBPLS exhibited a superior performance to El-net Cox, RSF, Block Forest, and MCIA, having higher probability of selecting the correct (important) features (Figure 2). In Table 2, the range of the prediction performance (C-index) of the proposed Cox-sMBPLS model was 0.60–0.64, for El-net Cox was 0.49–0.51, for RSF was 0.50–0.53, for Block Forest was 0.49–0.51, and for MCIA was 0.48-0.51 for different changing parameters. There is a recent benchmark analysis (Jardillier et al., 2020) of lasso-like penalties (including ridge, lasso, adaptive lasso, and elastic-net) of the Cox model where the authors showed that the lasso-like penalized Cox model (including El-net Cox) have the potential of having ≥50% of false discovery proportion. This is consistent with our simulation results where El-net Cox performs poorly in most of the scenarios.

Overall, the proposed supervised Cox-sMBPLS method outperformed all competing methods (El-net Cox, RSF, Block Forest, and MCIA) regarding the exact survival prediction and feature-selection power. Moreover, this method showed less sensitivity to the selection of the tuning parameters and censoring rate compared to competing methods.

### Results on a Heart-Failure Discovery Cohort

**FIGURE 3:**
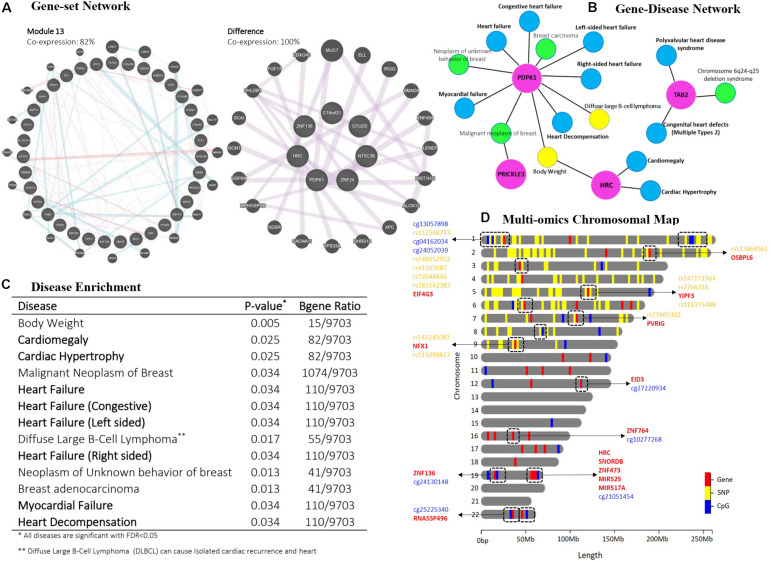
Post-hoc analyzes for multi-mics modules 10 and 13 resulted from the Cox-sMBPLS model. **(A)** gene-set network analysis. **(B)** disease ontology for *PDPK1*, *TAB2*, *PRICKLE3*, and *HRC* genes which are presented in both modules. **(C)** disease enrichment analysis. **(D)** chromosomal map. Dashed boxes show the Omics profiles, which are located in a small window on the same chromosome.

The supervised Cox-sMBPLS model (on HF cohort) retained *k* = 15 multi-Omics modules (i.e., a combination of genes, SNPs, and CpGs affecting the survival probability when occurring together). *k* is tuned using a 5-fold CV within the range of 1 = *k* = 73. The upper boundary (*k=73*) is calculated based on Chung et al. (2012) suggestion as $min\left\{p,\frac{\nu -1}{\nu }n\right\}$, where *p* is the total number of the covariates, ν is the fold number in the (k-fold) CV, and *n* is the sample size. Results are provided in Supplementary Section 2.3. Figure 3 presents two significant multi-Omics modules (10, 13) in the final Cox model (*p*-value < 0.1) in detail. Module 10 contains 375 features (33 genes, 308 SNPs, 34 CpGs) and module 13 contains 497 features (49 genes, 399 SNPs, 49 CpGs). There are 122 Omics profiles (16 genes, 91 SNPs, and 15 CpGs), included in module 13 but not module 10. Details for these 122 features are also included in Figure 3 (see also Supplementary Tables 4–6).

We also compared the prediction performance of the Cox-sMBPLS model to El-net Cox (Simon et al., 2011), RSF (Ishwaran et al., 2008), Block Forest (Hornung and Wright, 2019) and MCIA (Min and Long, 2020) (see Supplementary Table 7 for prediction performance measures). All models are trained on 80% of the samples and tested on the left-out 20% portion. The proposed supervised Cox-sMBPLS showed better performance regarding both C-index and AUC measures.

To interpret the biological relevance of the significant multi-Omics modules enrichment analysis of their Omics profiles using network-based resources and disease ontology is undertaken. Specifically, we performed a gene-set network analysis (Figure 3A) using GeneMANIA (Warde-Farley et al., 2010), gene-disease network (Figure 3B), and disease enrichment analysis (Figures 2D, 3C), both using DisGeNET knowledge platform (Piñero et al., 2020). Gene-set network analysis shows 82% and 100% co-expression between the genes in multi-Omics module 13 and the difference between modules 10 and 13, respectively. Modules 10 (*p* = 0.097) and 13 (*p* = 0.059) are the two significant modules (see Supplementary Table 4). There is a 61% decrease in P-value from module 10 to module 13 (from 0.097 to 0.059). To figure out the legitimacy of this strengthening in the resulted association (from module 10 to module 13), we removed the overlaps between these two modules and ran a gene-set network analysis for the remaining genes (which are causing this 61% decrease in P-value). The result showed 100% co-expression between them, which proves that this boost from module 10 to module 13 is biologically genuine and worthy to run further functional validations to study them for finding novel biomarkers. Moreover, gene-disease network analysis of the selected genes common in both modules (*PDPK1*, *TAB2*, *PRICKLE3*, and *HRC*) also confirms the role of these genes in heart complications. Disease enrichment results similarly show that the genes in the multi-Omics modules are mainly enriched for heart disease, such as heart failure, cardiac hypertrophy, and myocardial failure. A brief discussion of the common genes follows.

*PDPK1* (Phosphoinositide-dependent protein kinase-1) is the *PDK1* protein coding gene and also a part of the *AGC* super family of protein kinases which have been well documented for playing a crucial role in heart complications (Marrocco et al., 2019). It has also been reported as a component of the TGF-β/smad signaling pathway which leads to decompensation and heart failure (Kuzmanov et al., 2016). Histidine-rich calcium binding protein (*HRC*) can affect Ca2+ cycling in the sarcoplasmic reticulum (SR) that could cause the mitochondrial death pathway and enhance cardiac function in failure heart (Park et al., 2012). *TAB2* (*TAK1* binding protein-2) is known to play an important role in cardiac development and has recently received more attention in heart diseases. There has been recent research suggesting *TAB2* and its signaling network (*TAB2-TAK1*) as novel therapeutic targets in heart complications (Yin et al., 2017; Cheng et al., 2020). Moreover, a first report of a Chinese family with Congenital heart defects (CHD) caused by a novel *TAB2* nonsense mutation has been published in 2020 (Chen et al., 2020). We additionally tracked the multi-Omics profiles on a chromosomal map. Figure 3D shows the chromosomal map for module 13, indicating the combination of two or more different Omics profiles located within a small window on the same chromosome.

These follow-up analyses suggest the biological relevance of the multi-Omics modules resulted from the proposed Cox-sMBPLS algorithm. However, further functional and validation studies (such as *in vivo* validation using animal models) are required to identify novel biomarkers.

## Discussion

A survival prediction model (Cox-sMBPLS) based on leveraging and integrating information across multi-Omics compartments via the *cis*-regulatory quantitative effects (eQTL, eQTM, meQTL) was developed. It also enables identification of multi-Omics modules -combinations of different Omics features- exhibiting a large effect on survival probabilities. The proposed modeling framework can easily accommodate a large number of blocks and thus other Omics types with minor modifications.

In the past decade, a large body of literature was developed to introduce methods relating Omics profiles and time to an event such as recurrence in cancer patients, death, etc. Cox-PH (Cox, 1972) is the most widely used method to model the time to such events, for which several high-dimensional adaptations have been proposed in the literature (Park et al., 2002; Tan et al., 2006; Zhang and Zhang, 2020). To also leverage the biological information besides the censoring, we employed the *cis*-regulatory information and a censoring-reweighting technique in our proposed algorithm. The key output of the Cox-sMBPLS is to determine multi-Omics modules that are most associated with disease progression and patient survival.

Simulation studies showed that both the prediction and feature-selection performance of Cox-sMBPLS is significantly better than competing procedures (El-net Cox and RSF) across multiple settings (Tables 1, 2) and in a heart failure study (Figure 3). The gene-set enrichment and disease ontology results confirmed biological relevance of the identified multi-Omics modules. Particularly, we found *PDPK1* and *TAB2* associated with HF which have been well documented for playing a crucial role in heart complications (Kuzmanov et al., 2016; Yin et al., 2017; Marrocco et al., 2019; Chen et al., 2020; Cheng et al., 2020).

A direction of future research is to enhance the incorporation of additional prior biological knowledge; e.g., include functional pathway information. On the validation front, analysis of data from animal studies can assist in identifying novel non-coding features prioritized by significant multi-Omics modules.

## Data Availability Statement

The data analyzed in this study is subject to the following licenses/restrictions: Data are available from JD upon reasonable request. Requests to access these datasets should be directed to JD, juliod@cop.ufl.edu.

## Ethics Statement

The studies involving human participants were reviewed and approved by The University of Illinois at Chicago (UIC). The patients/participants provided their written informed consent to participate in this study.

## Author Contributions

NV and GM designed the study and wrote the manuscript. NV, CM, AD, LC, and JD performed the multi-Omics data preparation and quality control. NV and JD implemented the analysis. All authors read and approved the final manuscript.

## Conflict of Interest

The authors declare that the research was conducted in the absence of any commercial or financial relationships that could be construed as a potential conflict of interest. The reviewer HQ declared a shared affiliation with the authors NV, CM, LC, JD, and GM to the handling Editor at time of review.

## Publisher’s Note

All claims expressed in this article are solely those of the authors and do not necessarily represent those of their affiliated organizations, or those of the publisher, the editors and the reviewers. Any product that may be evaluated in this article, or claim that may be made by its manufacturer, is not guaranteed or endorsed by the publisher.

## Footnotes

1
https://www.rdocumentation.org/packages/survival/versions/2.11-4/topics/survfit

2
https://cran.r-project.org/web/packages/spls/index.html

## Supplementary Material

Supplementary Section 1 Supplementary Methods, including Cox-sMBPLS objective function recasting, and model performance measures.

Supplementary Section 2 Supplementary Results including simulation experiments, additional simulation results, and additional results for the heart failure data.
