# Supplementary material for: Cox-sMBPLS: An Algorithm for Disease Survival Prediction and Multi-Omics Module Discovery Incorporating Cis-Regulatory Quantitative Effects
Source: Front Genet. 2021 Aug 2;12:701405. doi: 10.3389/fgene.2021.701405 (PMC8366414; doi:10.3389/fgene.2021.701405)
Supplement: Supplementary Section 1 — Supplementary Methods, including Cox-sMBPLS objective function recasting, and model performance measures. [file Data_Sheet_1.pdf]

Supplementary Material for

Multi-Omics data integration using a supervised Cox-sMBPLS model incorporating prior biological information

S1 Supplementary Methods

S1.1 Cox-sMBPLS objective function recasting

In this subsection we provide a brief history of recasting a PCA-kind problem as a (ridge or elastic-net) regression that helps to understand equation (2). The original recasting (from eq(1) to eq(2)) comes from the regression approach to principal component analysis - PCA (Zou *et al.*, 2006) where the common PCA is re-formed in terms of a (ridge) regression, as follows:

$$\hat{\beta} = \arg min_{\beta} \|T_i - \boldsymbol{X}\beta\|^2 + \lambda\|\beta\|^2 + \lambda_1\|\beta\|_1,$$

where T_i is the ith principal component, PC ($i = 1, \dots, n$), $\boldsymbol{X}$ is the matrix of the covariates, β is the regression coefficients, λ is the scaling factor, and λ_1 is the $L_1 - penalty$. This problem is then solved using the LARS-EN algorithm (Zou and Hastie, 2005) for a fixed λ; and $X\boldsymbol{V}$ ($\boldsymbol{V} = \frac{\hat{\beta}}{\|\hat{\beta}\|}$) is considered as the ith approximate principle component (PC). They prove the following optimization problem as the sparce PCA criterion:

$$\left(\hat{\boldsymbol{A}}, \hat{\boldsymbol{B}}\right) = \arg min_{\boldsymbol{A},\boldsymbol{B}} \sum_{i=1}^{n} \|x_i - \boldsymbol{A}\boldsymbol{B}^T x_i\|^2 + \lambda \sum_{j=1}^{k} \left\|\beta_j\right\|^2 + \sum_{j=1}^{k} \lambda_{1,j} \left\|\beta_j\right\|_1, \;\; s.t. \acute{\boldsymbol{A}}\boldsymbol{A} = I_{k\times k}.$$

where k is the number of the latent components (PCs), x_i is the ith row-vector of the covariate matrix $\boldsymbol{X}$, $\boldsymbol{A} = [\alpha_1, \dots, \alpha_k]$, and $\boldsymbol{B} = [\beta_1, \dots, \beta_k]$. To minimize the above-mentioned sparse PCA criterion, a multi-step numerical solution is utilized as follows:

Step 1. Let $\boldsymbol{A}$ starts at $\boldsymbol{V}[, 1\!:k]$.

Step 2. Given a fixed $\boldsymbol{A}$, solve the (elastic-net) problem as:

$$\beta_j = \arg min_{\beta} \left(\alpha_j - \beta\right)^T \acute{\boldsymbol{X}}\boldsymbol{X}\left(\alpha_j - \beta\right) + \lambda\|\beta\|^2 + \lambda_{1,j}\|\beta\|_1.$$

Step 3. Given a fixed $\boldsymbol{B}$, compute the SVD of $\acute{\boldsymbol{X}}\boldsymbol{X}\beta = \boldsymbol{U}\boldsymbol{D}\acute{\boldsymbol{V}}$, and then update $\boldsymbol{A}$ as $\boldsymbol{A} = \boldsymbol{U}\acute{\boldsymbol{V}}.$

Step 4. Repeat steps 2-3 until convergence, and normalization $\hat{V}_j = \frac{\beta_j}{\|\beta_j\|}$ $(j = 1, \dots, k)$.

Thereafter, Chun and Keleş, 2010 proposed a sparse partial least square (PLS) formulation by generalizing the formulation of the sparse PCA (Zou *et al.*, 2006) as follows:

$$(\widehat{w}, \hat{c}) = \arg min_{w,c} \{-\kappa\, \acute{w}Mw + (1-\kappa)(c-w)'M(c-w) + \lambda_1|c|_1 + \lambda_2|c|_2^2\}, \qquad s.t. \acute{w}w = 1\,.$$

where $M = \acute{X}Y\acute{Y}X$, κ is the concave-penalty parameter to control the amount of the weight is given to the concave part of the objective function ($\acute{w}Mw$), and therefore, to control the local-solution issue. Hence, smaller κ can reduce the effect of the concave part. Chun and Keleş, 2010 showed, via a simulation study, that $0 < \kappa < \frac{1}{2}$ might lead to a numerically easier optimization problem. However. It is proven that for univariate response variable (such as survival data) the solution of objective function does not depend on the value of κ. The original idea of concave-penalty comes from a direct formulation for sparse PCA using semidefinite programming, introduced by d'Aspremont *et al.*, 2005, to revisit the convexity issue in standard PCA.

The proof of the solution to problem in equation (2) is provided in (Chun and Keleş, 2010) for one-block data. Here, we re-write the theorem and its proof for the multi-block case.

Theorem S1. For univariate $\boldsymbol{y}$, the solution of problem (3) is $\hat{c}^{(b)} = \left(\frac{X^{\acute{(b)}}\boldsymbol{y}}{\left\|X^{\acute{(b)}}\boldsymbol{y}\right\|} - \frac{\lambda}{2}\right)_+ sign\left(\frac{X^{\acute{(b)}}\boldsymbol{y}}{\left\|X^{\acute{(b)}}\boldsymbol{y}\right\|}\right)$ where

$Z^{(b)} = \frac{X^{\acute{(b)}}\boldsymbol{y}}{\left\|X^{\acute{(b)}}\boldsymbol{y}\right\|}$ is the first direction vector of PLS.

Proof. For a given $c^{(b)}$ and $\kappa = 0.5$, it follows that $\widehat{w^{(b)}} = Z^{(b)}$ since the singular value decomposition of $Z^{(b)}Z\acute{^{(b)}}c^{(b)}$ yields $U^{(b)} = Z^{(b)}$ and $V^{(b)} = 1$. For a given $c^{(b)}$ and $0 < \kappa < 0.5$, the solution is given by $\widehat{w^{(b)}} = \left\{\frac{Z^{(b)\prime}c^{(b)}}{\|Z^{(b)}\|^2+\lambda^*}\right\}Z^{(b)}$ by using the Woodbury formula(Golub and Van Loan, 1983). Noting that $\frac{Z^{(b)\prime}c^{(b)}}{\|Z^{(b)}\|^2+\lambda^*}$ is a scalar and by the norm constraint, we have $\widehat{w^{(b)}} = Z^{(b)}$. Since the estimate of $w^{(b)}$ does not depend on $c^{(b)}$, we have $\hat{c}^{(b)} = \left(\frac{X\acute{^{(b)}}\boldsymbol{y}}{\|X\acute{^{(b)}}\boldsymbol{y}\|} - \frac{\lambda_1}{2}\right)_+ sign\left(\frac{X\acute{^{(b)}}\boldsymbol{y}}{\|X\acute{^{(b)}}\boldsymbol{y}\|}\right)$ for large λ2 (consider $\lambda = \lambda_1$).

S1.2 Model performance measures

As defined in section 2.1, Let $\tilde{y}_i$ and C_i indicate the true survival time and censoring time for the ith subject ($i = 1, \ldots, n$), respectively. The observed data consist of the pairs $\{(y_i, \delta_i)| \; i = 1, \ldots, n\}$ where $y_i = min(\tilde{y}_i, C_i)$ is the observed survival time and $\delta_i = I(\tilde{y}_i \le C_i)$ is the failure indicator. Note that $\tilde{y}_i = y_i$ if and only if $\delta_i = 1$ (i.e., the subject is uncensored). Suppose $\boldsymbol{Z} = \boldsymbol{X\acute{}\beta}$ is the linear predictor for survival probability (also called marker or risk-score), where $\boldsymbol{X}$ is the matrix of covariates with their corresponding coefficients $\boldsymbol{\beta}$**.** Therefore, the cumulative sensitivity, dynamic specificity, and the resulting cumulative/dynamic (C/D) AUC at time point t are defined as (Heagerty and Zheng, 2005; Kamarudin *et al.*, 2017):

$$Se_t(c) = P(\boldsymbol{Z}_i > c|y_i \le t),$$

$$Sp_t(c) = P(\boldsymbol{Z}_i \le c|y_i > t),$$

$$AUC(t) = P\big(\boldsymbol{Z}_i > \boldsymbol{Z}_j|y_i \le t, y_j > t\;\big) = \frac{P\big(\boldsymbol{Z}_i > \boldsymbol{Z}_j, \delta_i(t) = 1, \delta_j(t) = 0\big)}{P(\delta_i(t) = 1)\, P\big(\delta_j(t) = 0\big)}, \quad i \ne j.$$

Sensitivity measures the expected ratio of subjects with a risk-score value ($\boldsymbol{Z}_i$) greater than c among the subsample of uncensored individuals who died at time t. Specificity measures the expected ratio of subjects with a risk-score value ($\boldsymbol{Z}_i$) less than c among the subsample of censored individuals who survived beyond time

t. Direct estimation of $AUC(t)$ is challenging since we do not know the exact event time for the right-censored data.

Chambless and Diao, 2006, proposed an estimator by calculating $AUC(t)$ recursively using the risk sets at each event time-point. Suppose $t_1 < t_2 < \cdots < t_n$ are ordered event times. Therefore, the sensitivity, specificity and AUC at time point t_m ($1 \leq m \leq n$) are defined as:

$$Se_{t_m}(c) = P(\boldsymbol{Z}_i > c|\delta_i(t_m) = 1) = \frac{\sum_{k=1}^{m} \rho_k(c)\lambda(t_k)\, S(t_{k-1})}{1 - S(t_m)},$$

$$Sp_{t_m}(c) = P(\boldsymbol{Z}_i \leq c|\delta_i(t_m) = 0) = P(\boldsymbol{Z}_i \leq c) - \frac{\sum_{k=1}^{m}\big(1 - \rho_k(c)\big)\lambda(t_k)\, S(t_{k-1})}{S(t_m)},$$

$$AUC(t_m) = \frac{\left[\sum_{k=1}^{m} \gamma_k \lambda(t_k)\big(1 - \lambda(t_k)\big)S(t_{k-1})^2 - \sum_{k=1}^{m} \tau_k \lambda(t_k)\ \times\ \big(1 - S(t_{k-1})\big)S(t_{k-1})\right]}{S(t_m)\big(1 - S(t_m)\big)}, \qquad k \leq m.$$

where S and λ are survival and hazard functions (to be estimated by Kaplan-Meier using only the observed times of events), respectively, $t_0 = 0$, $\tau_0 = 0$, and

$$\gamma_k = P\big(\boldsymbol{Z}_i > \boldsymbol{Z}_j|\delta_i(t_k) = 1, \delta_i(t_{k-1}) = 0, \delta_j(t_k) = 0\big),$$

$$\tau_k = P\big(\boldsymbol{Z}_i > \boldsymbol{Z}_j|\delta_i(t_{k-1}) = 1, \delta_j(t_{k-1}) = 0, \delta_j(t_k) = 1\big),$$

$$\rho_k(c) = P(\boldsymbol{Z}_i > c|\delta_i(t_k) = 1, \delta_i(t_{k-1}) = 0), \qquad k \leq m.$$

Another estimator is introduced by Uno *et al.*, 2007, based on the inverse probability of censoring weighting. It modifies the naïve estimator by adding weights to the observed markers and time in a subsample of uncensored individuals. The weights are the probabilities of being uncensored when calculating the sensitivity. Therefore, the Uno's time-dependent sensitivity and specificity functions are defined as follows:

$$\widehat{Se}_t(c) = \frac{\sum_{i=1}^{n} I(\boldsymbol{Z}_i > c,\ y_i \le t)\left[\frac{\delta_i}{n\hat{S}_c(\,y_i)}\right]}{\sum_{i=1}^{n} I(\,y_i \le t)\left[\frac{\delta_i}{n\hat{S}_c(\,y_i)}\right]},$$

$$\widehat{Sp}_t(c) = \frac{\sum_{i=1}^{n} I(\boldsymbol{Z}_i \le c,\ y_i \le t)}{\sum_{i=1}^{n} I(\,y_i > t)}.$$

We also used the incident/dynamic (I/D) ROC-curves (Heagerty and Zheng, 2005) using the incident sensitivity $Se_t^I(c) = P\big(\acute{\boldsymbol{X}}\boldsymbol{\beta} > c|\delta(t) = 1\big)$ and dynamic specificity. Let us define the true-positive (TP) rate function as $TP_t(c) = Se_t(c)$, and false-positive (FP) rate function as $FP_t(c) = 1 - Sp_t(c)$. Therefore, the I/D ROC curve and the area under the ROC curve (AUC) can be formulated as $ROC_t(p) = TP_t\{[FP_t]^{-1}(p)\}$ and $AUC(y) = \int_0^1 ROC_t(p)dp$ (for $p \in [0,1]$).

S2 Supplementary Results

S2.1 Simulation experiments

We examined 9 different scenarios that varied the following components: k the number of latent components, δ censoring rate, b number of blocks, and p_b number of features in block b. Simulation setups are shown in Table 1. We sampled true predictor matrices X_T^b ($b = 1,2,3$) of dimension $n \times p_b$, with fixed sample size $n = 91$, and $p_b = 100\,\upsilon^{(b)}$, $p_b = 1000\,\upsilon^{(b)}$ and $p_b = 10\,000\,\upsilon^{(b)}$ to consider low, moderate and high levels of dimensionality, respectively. we defined $\upsilon^{(b)}$, as the weight of each block, relative to the total number of the genes: $\upsilon^{(1)} = 1$, $\upsilon^{(2)} = \frac{Total\ number\ of\ the\ SNPs}{Total\ number\ of\ the\ genes} = \frac{578\,846}{27\,645} = 20.9$, $\upsilon^{(3)} = \frac{Total\ number\ of\ the\ CpGs}{Total\ number\ of\ the\ genes} = \frac{12\,283}{27\,645} = 0.4$. Matrices X_T^b ($b = 1,2,3$) are random samples from the real-world Omics data with a total of 27 645 genes, 578 846 SNPs, and 12 283 CpGs (explained in section 3.2). Therefore, we will have the following number of features to be sampled from each Omics block in different dimensionality levels:

$$Low-dim\colon \begin{cases} p_1 = & 100 \times 1 & = 100 \\ p_2 = & 100 \times 20.9 & = 2090 \\ p_3 = & 100 \times 0.4 & = 40 \end{cases}$$

$$Moderate-dim: \begin{cases} p_1 = & 1000 \times 1 & = 1000 \\ p_2 = & 1000 \times 20.9 & = 20\ 900 \\ p_3 = & 1000 \times 0.4 & = 400 \end{cases}$$

$$High-dim: \begin{cases} p_1 = & 10\ 000 \times 1 & = 10\ 000 \\ p_2 = & 10\ 000 \times 20.9 & = 209\ 000 \\ p_3 = & 10\ 000 \times 0.4 & = 4\ 000 \end{cases}$$

For instance, in the moderate level of dimensionality, we randomly sampled 1000 genes (from the gene-expression data), 20 090 SNPs (from the genotype data), and 400 CpGs (from methylation data). For the construction of true latent components ($\boldsymbol{\tau}_T$), we assume that some of the features in each block have small or no effect on the response variable by specifying sparse (true) direction vectors ($\boldsymbol{w}_T$). Therefore, true latent components are sparse across all simulations. The response variable pair (y_i, δ_i) for sample i ($i = 1, \dots, 91$) is simulated using a flexible hazard model (Harden and Kropko, 2019) instead of employing a known distribution (such as Weibull or exponential). This results in a more generalizable survival data. Therefore, we used the *sim.survdata* function (*coxed* package), which generates the baseline hazard function by fitting a cubic-spline to random points. For each simulation scenario, $S = 900$ datasets are generated. Within each scenario, datasets are generated using three different k ($k = 2{,}5{,}10$), each with 300 replications. We also used six different seed numbers and the results assess stability of the models against the seed numbers. The simulation algorithm is described in Algorithm 2 (in the manuscript). In each iteration, we tuned the number of components (k) and the sparsity (λ) for the Cox-sMBPLS model, elastic-net penalty parameters (λ_1 and λ_2) for the El-net Cox model, and number of the tree (α_1), node size (α_2) and number of variables available for splitting at each tree node (α_3), also called *mtry*, for RSF.

S2.2 Additional simulation results

The simulation results for scenarios with a low level of dimensionality (total of 2230 features and 91 samples), a moderate level of dimensionality (total of 22 300 features and 91 samples) and a high level of dimensionality (total of 223 000 features and 91 samples) are reported in Tables S1 to S3. Figures S1 and S2 show the boxplots for **(A)** Harrell's C-index, **(B)** I/D AUC, **(C)** C/D AUC, and **(D)** Uno's AUC values. Results are shown for

different censoring rates ($\delta = 10,\ 40, 60\%$) and number of components ($k = 2,\ 5, 10$). Performance measures (C-index and AUCs) and their standard deviations (SDs) are averaged over $S = 450$ simulations for scenarios with low and moderate dimensionality, and $S = 300$ for the scenario with a high level of dimensionality. The prediction performance (C-index) and feature-selection performance (AUCs) of our proposed Cox-sMBPLS model remained higher than El-net Cox and RSF regardless of the different numbers of components ($k = 2, 5, 10$), censoring rates ($\delta = 10, 40, 60\%$). In the settings with a moderate level of dimensionality (Table S2), when increasing the censoring rate from 10% to 60%, the cumulative AUC (C/D) AUC of all models dropped down, except MCIA (which increased by 2%): in Cox-sMBPLS decreased by 2%, 4% and 4% for $k = 2{,}5{,}10$, respectively; in El-net Cox decreased 8%, 7% and 11% for $k = 2{,}5{,}10$, respectively; in RSF decreased by 11%, 12% and 10% for $k = 2{,}5{,}10$, respectively; in Block Forest decreased by 6%, 5% and 5% for $k = 2{,}5{,}10$, respectively. It shows that the proposed Cox-sMBPLS model is less sensitive to the censoring rate compare to other methods. Overall, our proposed supervised Cox-sMBPLS method outperformed the El-net Cox and RSF regarding the exact survival prediction and feature selection. Moreover, this method is less sensitive to the tuning parameters selection (including the number of the components, k) compared to the other methods.

Table S1. Simulation results for the scenarios with a low level of dimensionality (total of 2230 features and 91 samples). Performance measures (C-index and AUCs) and their standard deviations (SDs) are averaged over $S = 450$ simulations in each setting. Parameter k indicates the number of components. SDs are shown in the parentheses.

**Censoring %**	Measure	**Number of Components**														
		$k = 2$					$k = 5$					$k = 10$				
		Cox-sMBPLS	El-net Cox	RSF	Block Forest	MCIA	Cox-sMBPLS	El-net Cox	RSF	Block Forest	MCIA	Cox-sMBPLS	El-net Cox	RSF	Block Forest	MCIA
10% (Low)	C-index	0.60 (0.10)	0.49 (0.08)	0.50 (0.10)	0.49 (0.09)	0.51 (0.09)	0.60 (0.09)	0.51 (0.07)	0.53 (0.10)	0.51 (0.10)	0.51 (0.09)	0.62 (0.11)	0.50 (0.09)	0.51 (0.10)	0.49 (0.10)	0.51 (0.09)
	C/D AUC*	0.95 (0.13)	0.38 (0.30)	0.46 (0.06)	0.87 (0.13)	0.91 (0.12)	0.97 (0.09)	0.36 (0.29)	0.46 (0.09)	0.87 (0.12)	0.91 (0.12)	0.97 (0.09)	0.40 (0.28)	0.44 (0.11)	0.87 (0.13)	0.91 (0.12)
	I/D AUC**	0.58	0.57	0.58	0.57 (0.07)	0.57 (0.07)	0.58	0.57	0.57	0.57 (0.07)	0.57 (0.07)	0.60	0.57	0.58	0.58 (0.07)	0.57 (0.07)

		(0.07)	(0.06)	(0.07)			(0.08)	(0.07)	(0.08)			(0.10)	(0.08)	(0.07)		
	Uno's AUC***	0.52 (0.22)	0.46 (0.18)	0.48 (0.22)	0.46 (0.23)	0.49 (0.23)	0.53 (0.23)	0.44 (0.18)	0.46 (0.23)	0.46 (0.23)	0.45 (0.23)	0.53 (0.23)	0.48 (0.21)	0.49 (0.24)	0.48 (0.24)	0.45 (0.23)
40% (Moderate)	C-index	0.62 (0.11)	0.49 (0.09)	0.51 (0.10)	0.49 (0.10)	0.51 (0.11)	0.63 (0.11)	0.49 (0.09)	0.51 (0.10)	0.49 (0.11)	0.51 (0.11)	0.62 (0.10)	0.50 (0.10)	0.51 (0.10)	0.48 (0.11)	0.5 (0.11)
	C/D AUC	0.94 (0.18)	0.35 (0.27)	0.39 (0.19)	0.84 (0.19)	0.90 (0.20)	0.94 (0.17)	0.36 (0.27)	0.37 (0.19)	0.84 (0.19)	0.90 (0.20)	0.97 (0.09)	0.37 (0.27)	0.40 (0.17)	0.84 (0.19)	0.91 (0.20)
	I/D AUC	0.60 (0.08)	0.57 (0.07)	0.57 (0.08)	0.56 (0.07)	0.57 (0.07)	0.60 (0.07)	0.58 (0.07)	0.58 (0.08)	0.57 (0.07)	0.57 (0.07)	0.60 (0.09)	0.58 (0.07)	0.57 (0.07)	0.57 (0.06)	0.57 (0.07)
	Uno's AUC	0.54 (0.29)	0.44 (0.22)	0.45 (0.23)	0.44 (0.25)	0.42 (0.24)	0.56 (0.28)	0.45 (0.22)	046 (0.23)	0.45 (0.25)	0.42 (0.24)	0.55 (0.26)	0.45 (0.23)	0.45 (0.22)	0.44 (0.25)	0.42 (0.24)
60% (High)	C-index	0.63 (0.13)	0.50 (0.10)	0.51 (0.13)	0.49 (0.14)	0.48 (0.13)	0.64 (0.12)	0.50 (0.10)	0.50 (0.12)	0.50 (0.16)	0.48 (0.12)	0.63 (0.12)	0.50 (0.12)	0.51 (0.11)	0.48 (0.15)	0.48 (0.12)
	C/D AUC	0.93 (0.23)	0.30 (0.27)	0.35 (0.21)	0.81 (0.20)	0.93 (0.24)	0.93 (0.23)	0.29 (0.26)	0.34 (0.21)	0.82 (0.20)	0.93 (0.24)	0.93 (0.23)	0.29 (0.26)	0.34 (0.21)	0.82 (0.20)	0.93 (0.24)
	I/D AUC	0.61 (0.09)	0.58 (0.07)	0.59 (0.06)	0.57 (0.09)	0.58 (0.07)	0.60 (0.08)	0.59 (0.07)	0.59 (0.06)	0.61 (0.09)	0.58 (0.07)	0.61 (0.08)	0.60 (0.09)	0.60 (0.10)	0.60 (0.08)	0.59 (0.07)
	Uno's AUC	0.50 (0.31)	0.45 (0.24)	0.47 (0.27)	0.40 (0.28)	0.41 (0.29)	0.51 (0.32)	0.45 (0.24)	0.46 (0.27)	0.40 (0.30)	0.41 (0.29)	0.50 (0.32)	0.43 (0.23)	0.45 (0.25)	0.42 (0.30)	0.41 (0.27)

* Chambless estimator of cumulative/dynamic (C/D) AUC

** Incident/dynamic (I/D) AUC

*** Uno estimator of cumulative/dynamic (C/D) AUC

Table S2. Simulation results for the scenarios with a moderate level of dimensionality (total of 22 300 features and 91 samples). Performance measures (C-index and AUCs) and their standard deviations (SDs) are averaged over $S = 450$ simulations in each setting. Parameter k indicates the number of components. SDs are shown in the parentheses.

Censoring %	Measure	Number of Components														
		$k = 2$					$k = 5$					$k = 10$				
		Cox-sMBPLS	El-net Cox	RSF	Block Forest	MCIA	Cox-sMBPLS	El-net Cox	RSF	Block Forest	MCIA	Cox-sMBPLS	El-net Cox	RSF	Block Forest	MCIA
10% (Low)	C-index	0.57 (0.07)	0.48 (0.08)	50 (0.09)	0.49 (0.09)	0.50 (0.10)	0.57 (0.07)	0.50 (0.09)	0.50 (0.10)	0.49 (0.10)	0.50 (0.10)	0.56 (0.07)	0.50 (0.07)	0.50 (0.10)	0.51 (0.10)	0.50 (0.10)
	C/D AUC*	0.93 (0.19)	0.39 (0.27)	0.43 (0.13)	0.88 (0.11)	0.92 (0.11)	0.95 (0.16)	0.41 (0.26)	0.44 (0.12)	0.88 (0.11)	0.92 (0.11)	0.95 (0.15)	0.40 (0.27)	0.45 (0.11)	0.87 (0.15)	0.91 (0.15)
	I/D AUC**	0.58 (0.08)	0.56 (0.07)	0.58 (0.08)	0.58 (0.07)	0.57 (0.08)	0.58 (0.08)	0.57 (0.11)	0.58 (0.08)	0.57 (0.08)	0.58 (0.08)	0.58 (0.07)	0.56 (0.07)	0.58 (0.07)	0.57 (0.08)	0.58 (0.08)
	Uno's AUC***	0.51 (0.23)	0.48 (0.18)	0.48 (0.23)	0.47 (0.19)	0.52 (0.20)	0.56 (0.22)	0.47 (0.18)	0.48 (0.12)	0.46 (0.21)	0.52 (0.20)	0.51 (0.23)	0.48 (0.18)	0.46 (0.23)	0.46 (0.18)	0.52 (0.19)
40% (Moderate)	C-index	0.58 (0.09)	0.50 (0.09)	0.50 (0.09)	0.49 (0.11)	0.49 (0.11)	0.60 (0.10)	0.50 (0.09)	0.50 (0.11)	0.50 (0.11)	0.50 (0.11)	0.59 (0.09)	0.50 (0.09)	0.49 (0.10)	0.52 (0.12)	0.50 (0.12)
	C/D AUC	0.93 (0.21)	0.32 (0.28)	0.39 (0.18)	0.85 (0.18)	0.92 (0.19)	0.93 (0.20)	0.36 (0.27)	0.39 (0.17)	0.87 (0.15)	0.92 (0.15)	0.92 (0.22)	0.33 (0.28)	0.40 (0.17)	0.87 (0.15)	0.91 (0.15)
	I/D AUC	0.58 (0.08)	0.57 (0.07)	0.57 (0.06)	0.57 (0.06)	0.58 (0.07)	0.59 (0.08)	0.58 (0.07)	0.58 (0.07)	0.58 (0.07)	0.58 (0.07)	0.59 (0.08)	0.58 (0.08)	0.58 (0.07)	0.58 (0.07)	0.58 (0.07)
	Uno's AUC	0.53 (0.26)	0.47 (0.20)	0.46 (0.20)	0.46 (0.25)	0.52 (0.26)	0.55 (0.27)	0.48 (0.19)	0.44 (0.23)	0.42 (0.23)	0.48 (0.26)	0.55 (0.26)	0.46 (0.19)	0.46 (0.22)	0.40 (0.22)	0.48 (0.25)
60% (High)	C-index	0.61 (0.12)	0.50 (0.11)	0.51 (0.12)	0.51 (0.14)	0.49 (0.13)	0.61 (0.12)	0.50 (0.11)	0.51 (0.12)	0.48 (0.13)	0.51 (0.14)	0.62 (0.09)	0.50 (0.10)	0.50 (0.12)	0.50 (0.13)	0.51 (0.13)
	C/D AUC	0.92 (0.25)	0.30 (0.28)	0.33 (0.22)	0.86 (0.17)	0.93 (0.23)	0.91 (0.27)	0.31 (0.28)	0.33 (0.22)	0.87 (0.07)	0.93 (0.24)	0.92 (0.26)	0.26 (0.28)	0.34 (0.21)	0.87 (0.17)	0.93 (0.23)
	I/D AUC	0.60 (0.08)	0.59 (0.13)	0.60 (0.09)	0.59 (0.06)	0.59 (0.08)	0.60 (0.08)	0.58 (0.13)	0.59 (0.07)	0.60 (0.07)	0.59 (0.08)	0.60 (0.08)	0.59 (0.12)	0.60 (0.09)	0.59 (0.06)	0.58 (0.08)
	Uno's AUC	0.53	0.47	0.43	0.38 (0.26)	0.48 (0.30)	0.51	0.44	0.42	0.44 (0.28)	0.47 (0.31)	0.53	0.46	0.41	0.37 (0.30)	0.46 (0.30)

(0.29)	(0.21)	(0.23)	(0.30)	(0.23)	(0.25)	(0.32)	(0.20)	(0.25)

* Chambless estimator of cumulative/dynamic (C/D) AUC

** Incident/dynamic (I/D) AUC

*** Uno estimator of cumulative/dynamic (C/D) AUC

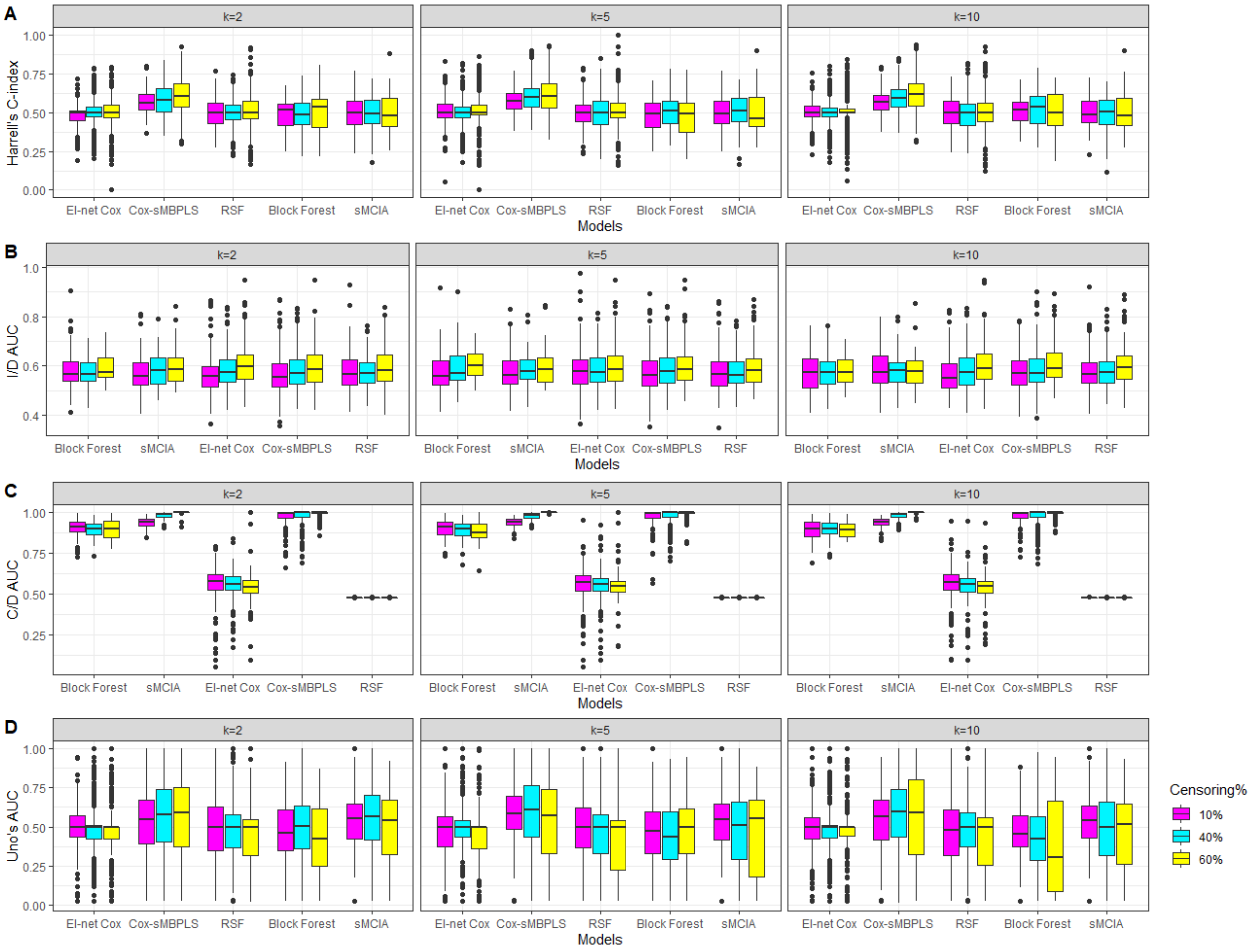

Fig. S1. Simulations results for scenarios with a <u>moderate</u> level of dimensionality (scenarios 2, 3, and 8). Boxplots for **(A)** Harrell's C-index, **(B)** I/D AUC, **(C)** C/D AUC, and **(D)** Uno's AUC values. Results are shown for different censoring rates ($\delta = 10,\ 40, 60\%$) and number of components ($k = 2,\ 5, 10$). Measures are averaged over $S = 300$ simulations.

Table S3. Simulation results for the high level of dimensionality (total of 2 230 00 features). Prediction performance measures (C-index and AUCs) and their standard deviations (SDs) are averaged over $S = 300$ simulations in each setting. Parameter k indicates the number of components. SDs are shown in the parentheses.

**Censoring %**	Measure	**Number of Components** $k = 2$			$k = 5$			$k = 10$		
		Cox-sMBPLS	El-net Cox	RSF	Cox-sMBPLS	El-net Cox	RSF	Cox-sMBPLS	El-net Cox	RSF
10% (Low)	C-index	0.61 (0.11)	0.49 (0.09)	0.50 (0.09)	0.61 (0.10)	0.48 (0.08)	0.50 (0.10)	0.61 (0.10)	0.50 (0.09)	0.51 (0.09)
	C/D AUC*	0.96 (0.10)	0.40 (0.25)	0.47 (0.07)	0.97 (0.09)	0.40 (0.25)	0.47 (0.06)	0.95 (0.13)	0.41 (0.26)	0.45 (0.10)
	I/D AUC**	0.57 (0.07)	0.56 (0.07)	0.57 (0.07)	0.58 (0.08)	0.57 (0.08)	0.27 (0.08)	0.59 (0.08)	0.57 (0.08)	0.58 (0.09)
	Uno's AUC***	0.49 (0.20)	0.47 (0.18)	0.48 (0.18)	0.50 (0.22)	0.50 (0.18)	0.47 (0.19)	0.49 (0.21)	0.48 (0.17)	0.47 (0.18)
40% (Moderate)	C-index	0.62 (0.12)	0.50 (0.09)	0.50 (0.10)	0.63 (0.12)	0.49 (0.08)	0.50 (0.11)	0.62 (0.13)	0.50 (0.08)	0.50 (0.11)
	C/D AUC	0.95 (0.17)	0.37 (0.27)	0.40 (0.18)	0.96 (0.13)	0.33 (0.28)	0.40 (0.17)	0.96 (0.13)	0.33 (0.28)	0.40 (0.18)
	I/D AUC	0.58 (0.08)	0.58 (0.09)	0.59 (0.09)	0.59 (0.08)	0.58 (0.07)	0.58 (0.07)	0.58 (0.07)	0.57 (0.07)	0.58 (0.08)
	Uno's AUC	0.46 (0.25)	0.48 (0.19)	0.46 (0.21)	0.49 (0.25)	0.49 (0.19)	0.46 (0.23)	0.47 (0.23)	0.47 (0.19)	0.47 (0.22)
60% (High)	C-index	0.63 (0.14)	0.50 (0.10)	0.51 (0.12)	0.63 (0.14)	0.49 (0.12)	0.49 (0.13)	0.64 (0.14)	0.51 (0.10)	0.49 (0.13)
	C/D AUC	0.94 (0.22)	0.29 (0.28)	0.33 (0.22)	0.94 (0.22)	0.33 (0.28)	0.34 (0.21)	0.95 (0.21)	0.29 (0.29)	0.34 (0.21)
	I/D AUC	0.60	0.59	0.60	0.59	0.59	0.60	0.59	0.59	0.59

		(0.09)	(0.08)	(0.08)	(0.08)	(0.08)	(0.09)	(0.08)	(0.08)	(0.08)
	Uno's AUC	0.43	0.44	0.43	0.44	0.43	0.45	0.45	0.45	0.45
		(0.30)	(0.22)	(0.23)	(0.29)	(0.23)	(0.24)	(0.28)	(0.21)	(0.23)

* Chambless estimator of cumulative/dynamic (C/D) AUC

** Incident/dynamic (I/D) AUC

*** Uno estimator of cumulative/dynamic (C/D) AUC

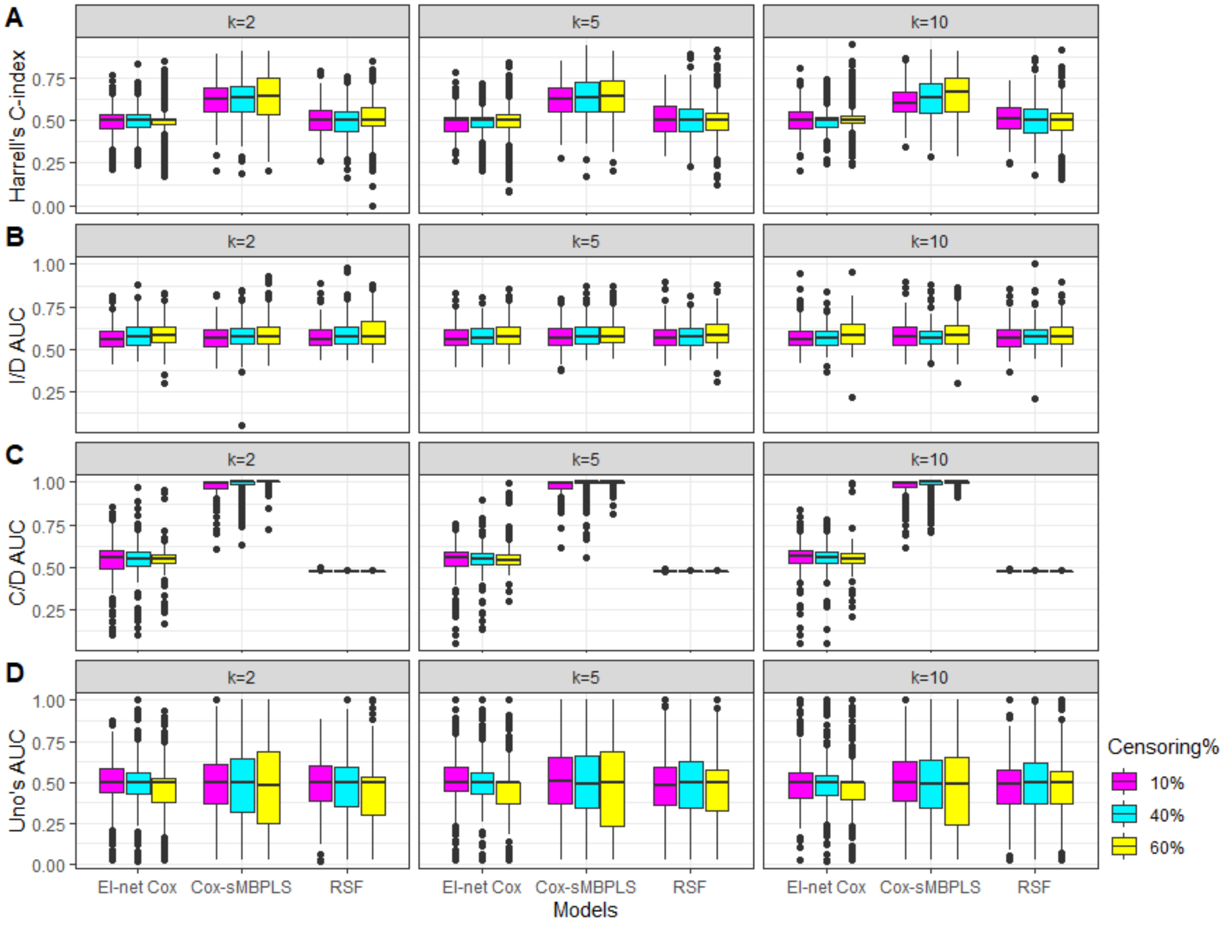

Fig. S2. Simulations results for scenarios with a high level of dimensionality (scenarios 2, 3, and 8). Boxplots for **(A)** Harrell's C-index, **(B)** I/D AUC, **(C)** C/D AUC, and **(D)** Uno's AUC values. Results are shown for different censoring rates ($\delta = 10,\ 40, 60\%$) and number of components ($k = 2,\ 5, 10$). Measures are averaged over $S = 300$ simulations.

S2.3 Additional results for the heart failure data

The supervised Cox-sMBPLS model retained $k = 15$ multi-Omics modules (i.e., a combination of genes, SNPs, and CpGs affecting the survival probability when occurring together). Table S4 shows the coefficients and p-values of these 15 modules based on the final Cox model. Table S5 shows the list of the Omics profiles included in module 13 but not module 10. These features almost double the significance of module 13 (p-value=0.059) compare to module 10 (p-value=0.097); i.e., they can be potential biomarkers for more investigations, such as functional validation. Table S6 shows the block importance which are the weight (importance) of each block in the supervised Cox-sMBPLS algorithm when calculating the features-weights and latent components.

Finally, the result of the model comparison and prediction performance of the proposed supervised Cox-sMBPLS, El-net Cox, RSF, Block Forest and MCIA models are presented in Table S7. The proposed supervised Cox-sMBPLS showed better prediction performance (higher C-index), and feature selection performance (higher AUCs) compared to El-net Cox and RSF model.

Table S4. Final results of the supervised Cox-sMBPLS model. The model retained $k = 15$ multi-Omics modules.

Multi-omics Module #	Coefficient	Standard Error	P-value
Module 1	8.66E-02	1.09E+01	0.994
Module 2	7.90E+03	7.79E+03	0.310
Module 3	4.36E+03	7.01E+03	0.534
Module 4	6.86E+02	2.94E+03	0.815
Module 5	2.60E+03	1.93E+03	0.178
Module 6	-2.35E+03	2.04E+03	0.249
Module 7	-1.58E+03	2.48E+03	0.523
Module 8	-9.89E+02	2.93E+03	0.736
Module 9	4.14E+03	2.66E+03	0.120
Module 10	-2.22E+03	1.34E+03	**0.097***
Module 11	-2.10E+02	1.13E+03	0.853
Module 12	-2.72E+02	5.61E+02	0.628
Module 13	-2.15E+03	1.14E+03	**0.059***
Module 14	-7.35E+02	7.74E+02	0.343

Module 15	1.92E+02	8.13E+02	0.814

* Significant with a p-value less than 0.1

Table S5. List of the Omics profiles included in module 13 but not module 10. Features are ordered based on their absolute weight in predicting survival probabilities. SNP names are formatted as “chromosome number.location”.

Component #	Feature Type	Feature Name	Feature Weight (absolute value)
13	Gene	ZNF24	0.142
13	Gene	MSX2P1	0.138
13	Gene	MIR517A	0.125
13	CpG	cg23712018	0.116
13	Gene	OTTHUMG00000015822	0.106
13	Gene	MIR545	0.097
13	CpG	cg25375711	0.097
13	Gene	HRC	0.095
13	CpG	cg05712903	0.094
13	Gene	NT5C3B	0.092
13	CpG	cg26730543	0.092
13	CpG	cg03973191	0.084
13	Gene	SNORD6	0.079
13	Gene	OTUD5	0.079
13	CpG	cg07382347	0.074
13	Gene	SNORD35B	0.067
13	Gene	OTTHUMG00000067216	0.064
13	Gene	LINC00674	0.063
13	CpG	cg06249604	0.061
13	CpG	cg18995788	0.055
13	CpG	cg14316629	0.055
13	Gene	C18orf21	0.046
13	SNP	X19.16151604	0.046
13	Gene	ZNF136	0.046
13	Gene	PDPK1	0.042
13	SNP	X9.18877268	0.038

13	SNP	X2.46125903	0.032
13	SNP	X12.29547807	0.030
13	CpG	cg10277268	0.028
13	CpG	cg25821399	0.027
13	SNP	X14.57795375	0.027
13	CpG	cg09196959	0.025
13	CpG	cg27565938	0.025
13	CpG	cg10568066	0.023
13	SNP	X12.72429431	0.017
13	SNP	X20.50324413	0.016
13	SNP	X11.61685791	0.010
13	SNP	X11.7931303	0.010
13	SNP	X7.148960867	0.010
13	SNP	X19.38758610	0.008
13	CpG	cg01286685	0.008
13	SNP	X10.105935654	0.007
13	SNP	X10.128221336	0.007
13	SNP	X17.17105786	0.007
13	SNP	X17.71760874	0.007
13	SNP	X3.169321429	0.007
13	SNP	X5.1160966	0.007
13	SNP	X13.42736047	0.006
13	SNP	X4.157366415	0.006
13	SNP	X16.84068409	0.006
13	SNP	X17.59470630	0.006
13	SNP	X22.36603179	0.006
13	SNP	X16.86553675	0.005
13	Gene	MIR525	0.004
13	SNP	X10.101340140	0.004
13	SNP	X10.97188433	0.004
13	SNP	X10.98517301	0.004
13	SNP	X12.68041846	0.004
13	SNP	X14.24551567	0.004
13	SNP	X17.7313498	0.004
13	SNP	X10.64270702	0.003

13	SNP	X13.103086368	0.003
13	SNP	X14.39349389	0.003
13	SNP	X19.55557550	0.003
13	SNP	X2.48966518	0.003
13	SNP	X20.1600405	0.003
13	SNP	X5.8503487	0.003
13	SNP	X10.28127690	0.003
13	SNP	X12.104778285	0.003
13	SNP	X2.242062825	0.003
13	SNP	X2.44311676	0.003
13	SNP	X2.75800568	0.003
13	SNP	X4.74717158	0.003
13	SNP	X8.75757172	0.003
13	SNP	X10.12596944	0.002

Table S6. Block importance. This table shows the weight (importance) of each block/module in the supervised Cox-sMBPLS algorithm when calculating the features-weights and latent components.

**Module #**	**Block**		
	**mRNA expression**	**Genotypes**	**DNA methylation**
**Module 1**	0.009	0.999	0.031
**Module 2**	0.010	0.004	0.013
**Module 3**	0.008	0.011	0.022
**Module 4**	0.024	0.008	0.037
**Module 5**	0.034	0.038	0.026
**Module 6**	0.009	0.052	0.017
**Module 7**	0.017	0.033	0.022
**Module 8**	0.011	0.033	0.022
**Module 9**	0.017	0.072	0.026
**Module 10**	0.014	0.029	0.065
**Module 11**	0.029	0.093	0.034
**Module 12**	0.018	0.049	0.111
**Module 13**	0.021	0.077	0.017

**Module 14**	0.022	0.078	0.018
**Module 15**	0.040	0.099	0.043

Table S7. Prediction performances for three models fitted on a real-world multiple -omics data. Data are collected from n=91 HF patients with 26 379 mRNA expression, 578 856 genotype and 12 283 DNA methylation profiles. Data is split to train-set (85%) and test-set (15%). All the performance measures are calculated using the test-set. Hyperparameters are tuned using CV.

Model	Tuned Hyperparameters	Test C-index	AUC	Uno's AUC*
**Supervised Cox-sMBPLS**	$\lambda = 0.95$ (soft threshold)<br>$k = 15$ (number of components)	0.75	97.3%	91.1%
**El-net Cox**	$\lambda_1 = 0.69$ (L_1 penalty)<br>$\lambda_2 = 0.81$ (L_2 penalty)	0.52	53.6%	28.8%
**RSF**	$\alpha_1 = 413$ (number of trees)<br>$\alpha_2 = 9$ (node size)<br>$\alpha_3 = 578$ (mtry**)	0.64	84.1%	77.4%
**Block Forest**	$\alpha_1 = 413$ (number of trees)	0.59	79.3%	75.2%
**MCIA** (sparse multiple co-inertia analysis)	-	0.61	80.1%	85.7%

* Uno's estimator of dynamic AUC for survival data

** Number of variables available for splitting at each tree node

Supplementary References

Chambless, L. E., & Diao, G. (2006). Estimation of time-dependent area under the ROC curve for long-term risk prediction. *Stat. Med., 25*(20), 3474-3486.

Chun, H., & Keleş, S. (2010). Sparse partial least squares regression for simultaneous dimension reduction and variable selection. *Journal of the Royal Statistical Society: Series B (Statistical Methodology), 72*(1), 3-25.

d'Aspremont, A., Ghaoui, L. E., Jordan, M. I., & Lanckriet, G. R. (2005). *A direct formulation for sparse PCA using semidefinite programming.* Paper presented at the Advances in neural information processing systems.

Harden, J. J., & Kropko, J. (2019). Simulating duration data for the cox model. *Political Science Research and Methods, 7*(4), 921-928.

Heagerty, P. J., & Zheng, Y. (2005). Survival model predictive accuracy and ROC curves. *Biometrics, 61*(1), 92-105.

Kamarudin, A. N., Cox, T., & Kolamunnage-Dona, R. (2017). Time-dependent ROC curve analysis in medical research: current methods and applications. *BMC Med. Res. Methodol., 17*(1), 53.

Uno, H., Cai, T., Tian, L., & Wei, L.-J. (2007). Evaluating prediction rules for t-year survivors with censored regression models. *Journal of the American Statistical Association, 102*(478), 527-537.

Zou, H., & Hastie, T. (2005). Regularization and variable selection via the elastic net. *Journal of the royal statistical society: series B (statistical methodology), 67*(2), 301-320.

Zou, H., Hastie, T., & Tibshirani, R. (2006). Sparse Principal Component Analysis. *Journal of Computational and Graphical Statistics, 15*(2), 265-286. doi:10.1198/106186006x113430
